# Single-cell transcriptomic analysis identifies systemic immunosuppressive myeloid cells and local monocytes/macrophages as key regulators in polytrauma-induced immune dysregulation

**DOI:** 10.1038/s41413-025-00444-x

**Published:** 2025-07-07

**Authors:** Drishti Maniar, M. Cole Keenum, Casey E. Vantucci, Tyler Guyer, Paramita Chatterjee, Kelly Leguineche, Kaitlyn Cheung, Robert E. Guldberg, Krishnendu Roy

**Affiliations:** 1 Wallace H. Coulter Department of Biomedical Engineering, Georgia Institute of Technology and Emory University, Atlanta, GA USA; 2 Department of Bioengineering, University of Oregon, Eugene, OR USA; 3 Knight Campus for Accelerating Scientific Impact, University of Oregon, Eugene, OR USA; 4 Parker H. Petit Institute for Bioengineering and Bioscience, Georgia Institute of Technology, Atlanta, GA USA; 5 Marcus Center for Therapeutic Cell Characterization and Manufacturing, Georgia Institute of Technology, Atlanta, GA USA; 6 Department of Biomedical Engineering, Vanderbilt University, Nashville, TN USA; 7 Department of Pathology, Microbiology, and Immunology, Vanderbilt University Medical Center, Nashville, TN USA; 8 Department of Chemical and Biomolecular Engineering, Vanderbilt University, Nashville, TN USA

**Keywords:** Pathogenesis, Bone

## Abstract

Polytrauma with significant bone and volumetric muscle loss presents substantial clinical challenges. Although immune responses significantly influence fracture healing post-polytrauma, the cellular and molecular underpinnings of polytrauma-induced immune dysregulation require further investigation. While previous studies examined either injury site tissue or systemic tissue (peripheral blood), our study uniquely investigated both systemic and local immune cells at the same time to better understand polytrauma-induced immune dysregulation and associated impaired bone healing. Using single-cell RNA sequencing (scRNA-seq) in a rat polytrauma model, we analyzed blood, bone marrow, and the local defect soft tissue to identify potential cellular and molecular targets involved in immune dysregulation. We identified a trauma-associated immunosuppressive myeloid (TIM) cell population that drives systemic immune dysregulation, immunosuppression, and potentially impaired bone healing. We found CD1d as a global marker for TIM cells in polytrauma. In the local defect tissue, we observed *Spp1*^+^ monocytes/macrophages mediating inflammatory, fibrotic, and impaired adaptive immune responses. Finally, our findings highlighted increased signaling via *Anxa1-Fpr2* and *Spp1-Cd44* axes. This comprehensive analysis enhances our understanding of immune dysregulation-mediated nonunion following traumatic injury and provides biomarkers that could function as treatment targets.

## Introduction

Bone has a remarkable ability to regenerate after injury, but severe trauma to multiple musculoskeletal tissues, termed polytrauma, has a high incidence of nonunion. Despite advancements in acute trauma care that have improved immediate patient survival, these advances have been accompanied by late-emerging complications, including longer hospital stays, revision surgical procedures, limb amputations, and in some cases death.^1^ Severe polytraumatic injuries involving large volumetric loss of bone, muscle, and connective tissues not only disrupt the local cellular niches that orchestrate tissue repair but can also provoke extreme activation of the systemic immune system, leading to systemic immune dysregulation and immunosuppression (SIDIS).^2,3^ Thus, both systemic and local immune dysregulation contribute to the failure of coordinated tissue repair, underscoring the need to address immune responses across both compartments to improve clinical outcomes.

We have previously established a preclinical rat model of polytrauma that mimics severe musculoskeletal trauma and the associated local and systemic immune responses, facilitating the investigation of SIDIS.^4–6^ The observed hallmarks of SIDIS include decreased effector immune cells (i.e., T cells and macrophages) and increased immunosuppressive myeloid cells, identified as CD11b^+^ HIS48^+^ cells in our established rat polytrauma model.^6–8^ CD11b^+^ HIS48^+^ cells are consistently elevated following polytrauma and correlate with worsening immune dysregulation and poor bone healing outcomes, suggesting they may serve as predictive biomarkers or therapeutic targets.^5,7^ CD11b and HIS48 are broad granulocytic and monocytic cell surface markers, and therefore, CD11b^+^ HIS48^+^ cells comprise a heterogeneous population of myeloid cells. This cell population contains immune cells arrested in their immature and immunosuppressive phase following prolonged inflammation after polytrauma, and other myeloid populations necessary for different functions. There is limited understanding of immunosuppressive subpopulations within the CD11b^+^ HIS48^+^ cells, including their specific roles in post-polytrauma pathogenesis and the markers they express. These cells share characteristics with myeloid-derived suppressor cells (MDSCs), a heterogeneous myeloid population linked to immunosuppression identified in other disease contexts. Therefore, characterizing these cells is critical for enabling more precise targeting strategies and mitigating off-target effects, such as those observed in previous attempts to target the broader CD11b^+^ HIS48^+^ population.^5^

While characterizing the systemic immune responses is crucial, understanding the immune responses in local defect tissue following polytrauma is equally important. Unlike the systemic environment, the local site of injury can experience persistent inflammation in complicated outcomes, which negatively impacts bone healing.^9,10^ Innate immune cells, such as neutrophils and macrophages, have been shown to dysregulate inflammation and contribute to impaired bone healing after polytrauma; however, the cellular and molecular mechanisms underlying this process remain poorly understood.^11,12^ Furthermore, interactions between immune cells and tissue-resident cells (e.g., stromal) are essential for bone formation, repair, and remodeling after injury to avoid pathological fibrosis.^13^ Studies investigating the crosstalk between inflammatory cells and tissue-resident cells involved in bone healing are also needed.

Understanding systemic and local immune responses independently offers valuable tissue-specific insights following polytrauma, but integrating these findings through global network analysis is essential to reveal how systemic and local immune cells interact and influence each other. Previous studies have primarily focused on immune responses in either systemic tissues, such as blood and bone marrow, or in the fracture hematoma and local defect tissues.^14,15^ However, to our knowledge, no study has examined the coordinated global interactions between systemic and local immune cells in the context of polytrauma-induced immune dysregulation.

Single-cell RNA sequencing (scRNA-seq) has emerged as a powerful tool for unraveling the complexity of cellular diversity and interactions at single-cell resolution. This technique can elucidate the cellular and molecular mechanisms mediating early immune responses, especially in the first week following polytrauma, and how they may lead to chronic SIDIS and disruption of pro-regenerative local cell signaling. Using scRNA-seq and an in vivo polytrauma rodent model, we identified trauma immunosuppressive myeloid (TIM) cells within the CD11b^+^ HIS48^+^ myeloid population, which appear to play a key role in SIDIS and impaired bone healing. These TIM cells express genes related to immunosuppressive IL-4/IL-13 and IL-10 signaling and express known markers of MDSCs. Flow cytometry revealed an expansion of CD1d^+^ TIM cells following polytrauma, suggesting CD1d as a potential marker for their identification and therapeutic targeting. Additionally, we identified a *Spp1*^+^ monocyte/macrophage (Mono/Mac) cluster in the local defect tissue associated with dysregulated inflammatory, fibrotic, and adaptive immune responses. Cell-cell interactions further demonstrated how myeloid cells, particularly TIM and Mono/Mac populations, drive polytrauma-induced immune dysregulation via pathways such as *Anxa1-Fpr2* and *Spp1-Cd44*. Overall, our study provides systemic and local cellular and molecular targets for further functional analysis, offering a hypothesis-generating framework for future investigations into polytrauma healing.

## Results

### Experimental design and scRNA-seq pipeline

**Fig. 1:**
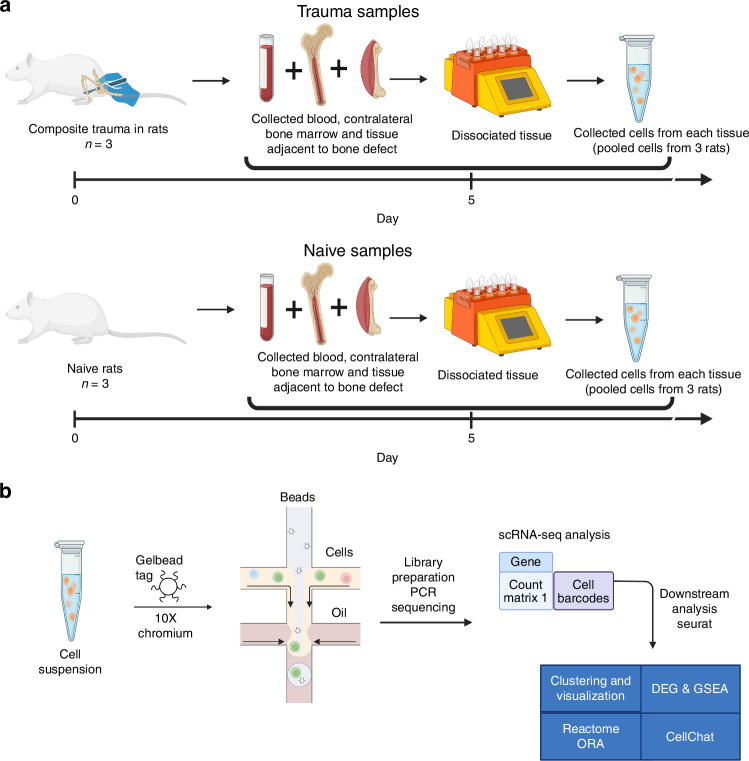
Experimental timeline of tissue collection from a rat polytrauma model and analytical overview of the single-cell RNA sequencing pipeline. **a** Polytrauma injuries were induced in three rats, and tissue collection was carried out on Day 5 post-injury. The collected tissues included blood, bone marrow, and the local defect tissue. Additionally, tissues from naïve animals with no injury were collected to serve as the control group. Following tissue collection, single cells were dissociated from each sample, and cells from the same tissue and condition were pooled for sequencing analysis. **b** The single-cell suspensions were tagged with DNA barcodes and processed using 10X Chromium Protocols. Subsequently, cDNA libraries were prepared by reverse transcription, PCR amplification, and sequencing. Alignment was performed using Cell Ranger, followed by gene matrix processing in R 4.2.2 with Seurat for clustering, visualization, and differential expression gene (DEG) analysis. Pathway analysis and post-DEG analysis were conducted using Reactome and fgsea packages. Ligand-receptor analysis was performed using CellChat

In this study, transcriptomic analysis was performed using a rat polytrauma model of bone nonunion and volumetric muscle loss. The cells from the blood, bone marrow, and the local defect tissue were isolated from naïve and trauma animals and prepared for scRNA-seq analysis (Fig. 1a). Using differentially expressed gene (DEG) analysis, Reactome over-representation analysis (ORA), gene set enrichment analysis (GSEA), and CellChat, we identified the pathways and cell types involved in polytrauma-induced immune dysregulation both systemically and at the local injury site (Fig. 1b).

### Major cell population of blood, bone marrow and local defect tissue

**Fig. 2:**
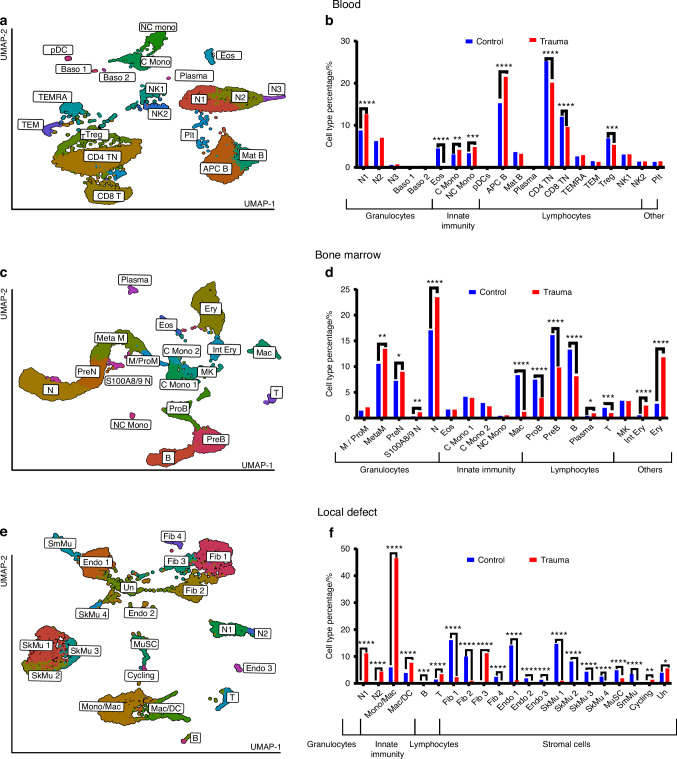
Single-cell RNA sequencing reveals expanded neutrophil, monocyte, and macrophage populations following polytrauma. Gene expression matrices from control and trauma groups were integrated and clustered via UMAP. Cell types were assigned based on canonical marker expression. **a** Blood UMAP identified innate and adaptive immune cell subsets. **b** Blood cell type percentages were compared across groups. **c** Bone marrow UMAP showed myeloid progenitors and B cell populations. **d** Bone marrow cell type percentages were visualized. **e** Local defect tissue UMAP revealed predominant stromal and immune cell populations. **f** Local defect tissue cell type percentages were compared. *P*-values were calculated by chi-squared test. ****P < 0.000 1, *P < 0.05; bar chart pairs without * were not significant. UMAPs were generated in Seurat v4; percentages plotted with Prism. N Neutrophils, Baso Basophils, Eos Eosinophils, C Mono Classical Monocytes, NC Mono Non-Classical Monocytes, pDC Plasmacytoid DCs, APC B Antigen-Presenting B Cells, Mat B Mature B Cells, Plasma Plasma Cells, CD4 TN Naive CD4 T Cells, CD8 T CD8 T Cells, TEMRA Effector Memory RA + CD8 T Cells, TEM Effector Memory CD8 T Cells, Treg Regulatory T Cells, NK Natural Killer Cells, Plt Platelets, M/ProM Myelocytes/Pro-Myelocytes, MetaM Metamyelocytes, PreN Pre-Neutrophils, Mac Macrophages, ProB Pro-B Cells, PreB Pre-B Cells, B B Cells, T T Cells, MK Megakaryocytes, Int Ery Intermediate Erythrocytes, Ery Erythrocytes, Mono/Mac Monocytes/Macrophages, Mac/DC Macrophages/Dendritic Cells, Fib Fibroblasts, Endo Endothelial Cells, SkMu Skeletal Muscle Cells, MuSC Muscle Stem Cells, SmMu Smooth Muscle Cells, Cycling Cycling Cells, Un Undetermined Cells

For each tissue, we performed clustering and cell type identification analysis of the combined polytrauma and naïve datasets using Seurat (Fig. 2a, c, e). Using integration on SCTransform normalized datasets, we identified the main cell types based on the expression of marker genes (Figs. S1–S3).^16–22^ Across the three tissues, various myeloid cells were identified, including neutrophils, monocytes (classical and non-classical), macrophages, eosinophils, and basophils. In the bone marrow, gene expression analysis differentiated neutrophil precursors into pre-neutrophils and myelocyte/promyelocytes. In the local defect tissue, monocytes, macrophages, and dendritic cells were challenging to distinguish and were categorized as Mono/Mac or Mac/DCs. Lymphocyte populations included naïve CD4 and CD8 T cells, regulatory T cells, CD8 effector memory T cells expressing CD45RA (TEMRA) and CD8 effector memory T cells (TEM) cells, natural killer cells, antigen-presenting B cells, and mature B cells. Stromal cells in the defect tissue included fibroblasts, skeletal and smooth muscle cells, endothelial cells, and muscle stem cells. An undefined cluster expressed genes linked to structural cells, like endothelial cells and fibroblasts.

The relative proportions of each cell type percentage in each tissue in polytrauma and control groups were compared (Fig. 2b, d, f). Most prominently, monocytes and macrophages in the blood and local defect were significantly increased in the polytrauma condition, and approximately half of all cells in the local defect after polytrauma were Mono/Mac or Mac/DC. Additionally, the results showed an increase in neutrophil subsets in the blood and local defect tissue in the polytrauma group compared to the control. This increase was accompanied by a predictable increase in the proportion of neutrophils and their precursors in the marrow. In the blood and bone marrow, these proportional increases were accompanied by relative decreases in the proportion of most T and B lymphocytes subtypes in the blood and bone marrow. In the local defect, the proportion of fibroblasts, endothelial cells, and muscle cells was largely decreased with polytrauma.

### Identification of systemic trauma immunosuppressive myeloid (TIM) cells

**Fig. 3:**
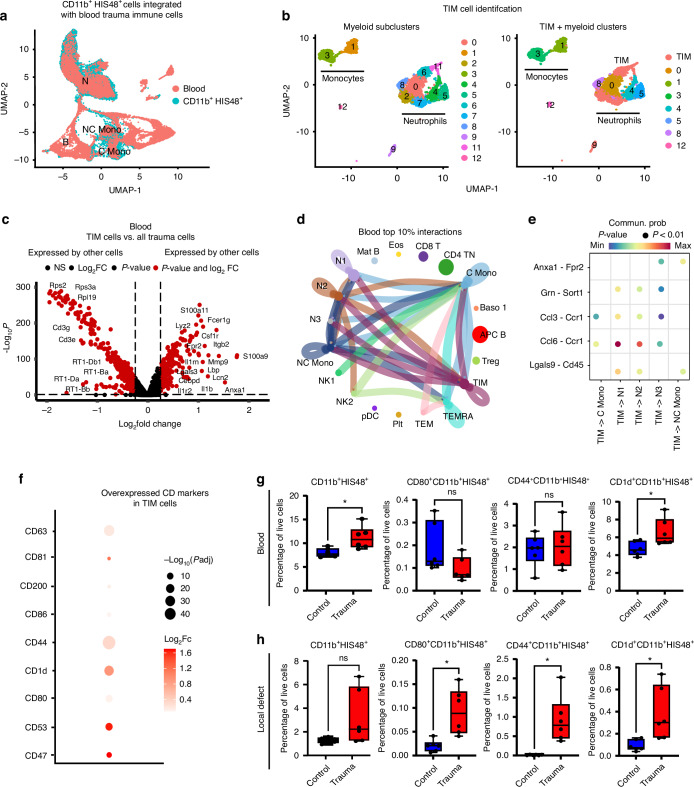
Systemic and local increase of CD1d^+^ TIM Cells and local increase in CD80^+^ and CD44^+^ TIM Cells implicate their role in polytrauma-induced immunosuppression. **a** Isolated CD11b^+^HIS48^+^ cells were integrated with blood immune cells to identify myeloid clusters with similar transcriptomic profiles to those of CD11b^+^HIS48^+^ cells as shown in the UMAP projections. **b** Clusters with high density expression of multiple of these immunosuppressive genes were identified as TIM cells. **c** DEGs of TIM cells compared to all polytrauma immune cells to identify TIM unique markers. The –Log_10_ (*P* values) indicates the level of significance of each gene while Log_2_ fold change represents the difference between the levels of expression for each gene between the polytrauma and control groups (baseline). **d** Chord diagrams show the most frequent ligand-receptor interactions where TIM cells in blood act as the signal source for myeloid cells. **e** Dot plots showing ligand-receptor interactions between TIM and other myeloid cells. **f** Cluster of differentiation (Cd) genes expressed by TIM cells. **g** Frequency of peripheral blood CD11b^+^HIS48^+^ or CD11b^+^HIS48^+^ cells expressing either TIM marker CD80, CD44, or CD1d. **h** Frequency of local defect CD11b^+^HIS48^+^ cells or CD11b^+^HIS48^+^ cells expressing either TIM marker CD80, CD44, or CD1d. Significance was determine using unpaired *t*-test or non-parametric Mann–Whitney test. Biological replicates *n* = 6, ****P < 0.000 1, ***P < 0.001, **P < 0.01, *P < 0.05

Given the role of circulating CD11b^+^HIS48^+^ cells in impaired bone healing, we aimed to better characterize this population and identify TIM cells in the blood. Since the target and gene associated with the HIS48 antibody are unknown, these populations could not be directly identified in transcriptomic datasets. To overcome this, CD11b^+^HIS48^+^ cells were isolated from the peripheral blood of polytrauma rats, sequenced, and independently integrated with blood polytrauma datasets. Post-integration analysis revealed that CD11b^+^HIS48^+^ cells clustered with all myeloid cells (monocytes and neutrophils) rather than forming a distinct myeloid cluster (Fig. 3a).

We refined our workflow for identifying TIM cells by isolating polytrauma myeloid cells, performing subclustering, and analyzing marker genes within these subclusters. Specifically, we focused on genes associated with immunosuppressive IL-4/IL-13 and IL-10 signaling and markers of MDSCs. Subclusters with high expression of multiple immunosuppressive genes were classified as TIM cells (Fig. S4). We identified 12 subclusters of myeloid cells of both the monocytic and granulocytic lineage from which to identify TIM cells (Fig. 3b, left panel). Based on their expression of immunosuppressive genes, clusters 2, 6, 7, 9, and 11 were identified as TIM cells while the remaining clusters represented other functional myeloid populations. All identified TIM clusters were composed of neutrophils, indicating these immunosuppressive cells are primarily of granulocytic rather than monocytic lineage (Fig. 3b, right panel). The classification was based on the genes associated with IL-4/IL-13 *(Anxa1, Itgax, Il2rg, Lcn2)*, IL-10 *(Ptafr, Csf1, Fpr1, Il1r2)*, or myeloid-derived suppressor cells *(Arg2, Il1b, Cd274, Nos2, Tgfb1)* (Figs. S5, S6).^23^ Clusters 9 and 11 expressed several genes associated with IL-4/IL-13 and IL-10 signaling and showed higher average expression of multiple immunosuppressive genes compared to all the other clusters (Fig. S5a, b). While clusters 2, 6, and 7 expressed a broad range of immunosuppressive genes similar to other clusters, they were selected as TIM cells due to their additional high expression of key immunosuppression-associated genes. For example, cluster 2 exhibited high expression of the IL-10-related, M2-phenotype-promoting gene *Csf1*,^24^ cluster 6 showed elevated expression of *Il1r2*, an antagonist of IL-1 signaling, and cluster 7 demonstrated high expression of *Nos2*, a gene involved in T cell dysregulation and immunosuppression (Fig. S5a–c).^25^ Overall, clusters 2,6,7 also showed high joint-density expression of these immunosuppressive genes (Fig. S6a–c). Through this comprehensive analysis, we identified the immunosuppressive subsets within the CD11b^+^HIS48^+^ population.

After identifying TIM cells, we determined their unique marker genes by performing DEG analysis between TIM cells and all other blood immune cells from the polytrauma group. This analysis revealed how the TIM cell transcriptomic profile distinctly differed from other immune cells under polytrauma conditions. TIM cells exhibited pronounced expression of key immunosuppressive genes, including *Anxa1, Il1r2, Il1rn*, and *Nos2* (Fig. 3c). These genes are known to dampen inflammatory responses and negatively impact T cell activity.^25–28^ Interestingly, in contrast to TIM cells, other immune cells were more expressive of several MHC-I genes (*RT1-Db1, RT1-Da, RT1-Ba, RT1-Bb*), which play a critical role in antigen presentation and the initiation of effective immune responses (Fig. 3c). The marker genes of TIM cells were associated with immunosuppressive pathways such as IL-4/IL-13 and IL-10 signaling, as well as increased ROS and RNS production (Fig. S7). Additionally, we compared the DEGs of TIM cells with control myeloid cells and observed a similar pattern, with TIM cells upregulating genes associated with IL-4/IL-13 and IL-10 signaling while downregulating MHC-I genes critical for antigen presentation to effector cells (Fig. S8). Overall, these results suggest that TIM cells express genes and activate pathways that significantly contribute to SIDIS, highlighting their critical role in polytrauma-induced immune dysregulation.

To understand how blood TIM cells influence SIDIS after polytrauma, we analyzed their ligand-receptor signaling interactions with other immune cells using CellChat. Analysis of the top 10% of ligand-receptor interactions in polytrauma blood revealed that TIM cells predominantly communicated with other myeloid cells (Fig. 3d). These interactions were predominantly driven by the *Ccl6-Ccr1* and *Anxa1-Fpr2* pathways, with the latter being a potent immunosuppressive signaling mechanism (Fig. 3e).^29^ These findings provide valuable mechanistic insights into how TIM cells interact with other immune cells and how they may contribute to the development of SIDIS after polytrauma.

Finally, we wanted to identify surface markers specific to TIM cells to enable their targeting following polytrauma. Since TIM cells represents an immunosuppressive subset within the broader CD11b^+^HIS48^+^ myeloid population, we used a two-step approach to identify and validate TIM surface markers within this population. First, we performed DEG analysis comparing TIM cells to control myeloid or other polytrauma immune cells, which identified several CD genes overexpressed by TIM cells including CD47, CD53, CD86, CD80, CD44, and CD1d (Fig. 3f). Second, we validated these markers by comparing their expression on different immune populations within the polytrauma condition using flow cytometry. While all six markers were detected on CD11b^+^HIS48^+^ cells, only CD80, CD44, and CD1d showed significantly higher expression on CD11b^+^HIS48^+^ cells compared to other HIS48^-^ immune cell populations in the polytrauma condition (Fig. S9a, b). These findings demonstrated that CD80, CD44, and CD1d could serve as specific markers to identify distinct TIM populations within the broader CD11b^+^HIS48^+^ myeloid population after polytrauma.

Having validated these markers in the polytrauma group, we next determined whether these TIM populations (CD80^+^CD11b^+^HIS48^+^, CD44^+^CD11b^+^HIS48^+^, and CD1d^+^CD11b^+^HIS48^+^) were enriched following injury by comparing their frequencies between naïve and polytrauma rats. We analyzed both blood and local defect tissue on Day 5 post-injury. In the blood, while the overall CD11b^+^HIS48^+^ population increased in polytrauma compared to controls (Fig. 3g, Fig. S10), only the CD1d^+^CD11b^+^HIS48^+^ TIM population showed a significant increase (Fig. 3g). In local defect tissue, although the total CD11b^+^HIS48^+^ population showed a non-significant increase in polytrauma (Fig. 3h, Fig. S10), all three TIM populations (CD80^+^CD11b^+^HIS48^+^, CD44^+^CD11b^+^HIS48^+^, and CD1d^+^CD11b^+^HIS48^+^) significantly increased in frequency (Fig. 3h). These findings reveal distinct patterns of TIM cell distribution. CD1d^+^CD11b^+^HIS48^+^ TIM cells expanded both systemically and locally in response to polytrauma, suggesting their broader role in polytrauma-induced immune dysregulation. In contrast, CD80^+^CD11b^+^HIS48^+^ and CD44^+^CD11b^+^HIS48^+^ TIM cells expanded specifically at the injury site, indicating a more localized function following polytrauma.

### The role of inflammatory Mono/Mac cells in the local defect tissue

**Fig. 4:**
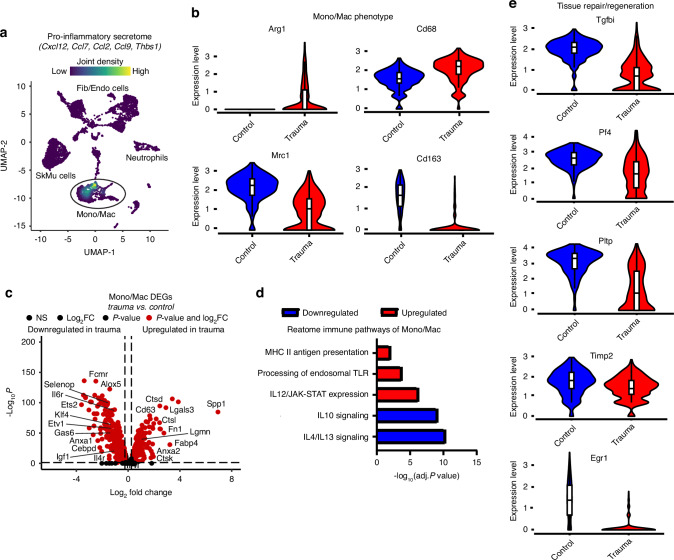
Mono/Mac cells exhibit pro-inflammatory and anti-reparative transcriptional responses in polytrauma. **a** Feature plot illustrating the expression of pro-inflammatory secretome genes within the Mono/Mac cluster. **b** Violin plots comparing the expression levels of Arg1, Cd68, Mrc1 (CD206), and Cd163 in the Mono/Mac cluster between control and polytrauma condition. **c** Differentially expressed genes (DEGs) in Mono/Mac cells, showing upregulated and downregulated genes in polytrauma compared to controls. The –Log_10_ (*P*-value) indicates the statistical significance of each gene, while Log_2_ fold change represents the magnitude of expression difference between conditions. **d** Over-representation analysis (ORA) of the top 200 upregulated and downregulated Mono/Mac DEGs identified significant immune pathways. Red represents upregulated pathways and blue downregulated pathways. Pathways were considered significant if their *P*-value was <0.01 and their false discovery rate (FDR) was <0.05. **e** Violin plots comparing the expression of genes associated with tissue repair and regeneration (Tgfbi, Pf4, Pltp, Egr1, Timp2) in the Mono/Mac cluster between control and polytrauma condition

Mono/Mac cells significantly increased in frequency under polytrauma condition compared to control within the local defect tissue (Fig. 2f). Therefore, Mono/Mac cells in the local defect tissue were analyzed to understand their role in chronic inflammation. These cells exhibited the highest expression of pro-inflammatory secretome genes (*Cxcl12*, *Ccl7*, *Ccl9*, *Thbs1*) relative to other myeloid and non-myeloid cells, highlighting their strong contribution to the inflammatory response after polytrauma (Fig. 4a). Despite systemic immunosuppression, the injury site may still experience chronic inflammation, hindering healing.^9^ Compared to controls, Mono/Mac cells in polytrauma upregulated genes such as *Arg1* and *Cd68*, markers of early and intermediate macrophages associated with inflammation, while downregulating genes linked to late-stage and repair macrophages, such as *Mrc1* (CD206) and *Cd163* (Fig. 4b).^14^ Differential gene expression analysis revealed an upregulation of pro-inflammatory genes, including *Spp1*, *Fabp4*, *Fn1*, and *Anxa2* (Fig. 4c), alongside activation of pro-inflammatory pathways like IL-12/JAK-STAT signaling and the macrophage activation pathway, MHC class II signaling (Fig. 4d). Conversely, immunosuppressive pathways such as IL-4/IL-13 and IL-10 signaling were downregulated, underscoring a heightened pro-inflammatory response with limited capacity to resolve inflammation (Fig. 4d). Furthermore, GSEA analysis revealed Mono/Mac cells downregulated pathways associated with the differentiation and activation of adaptive immune cells such as “T cell differentiation” and “B cell mediated immunity” (Fig. S11). Lastly, Mono/Mac cells decreased expression of tissue repair genes after polytrauma compared to control, including *Tgfbi, Pgf4, Pltp, Egr1, and Timp2* (Fig. 4e). These findings demonstrate that polytrauma reshapes the inflammatory response of Mono/Mac cells by amplifying inflammation and suppressing genes associated with immunosuppression, adaptive immune responses, and tissue repair.

**Fig. 5:**
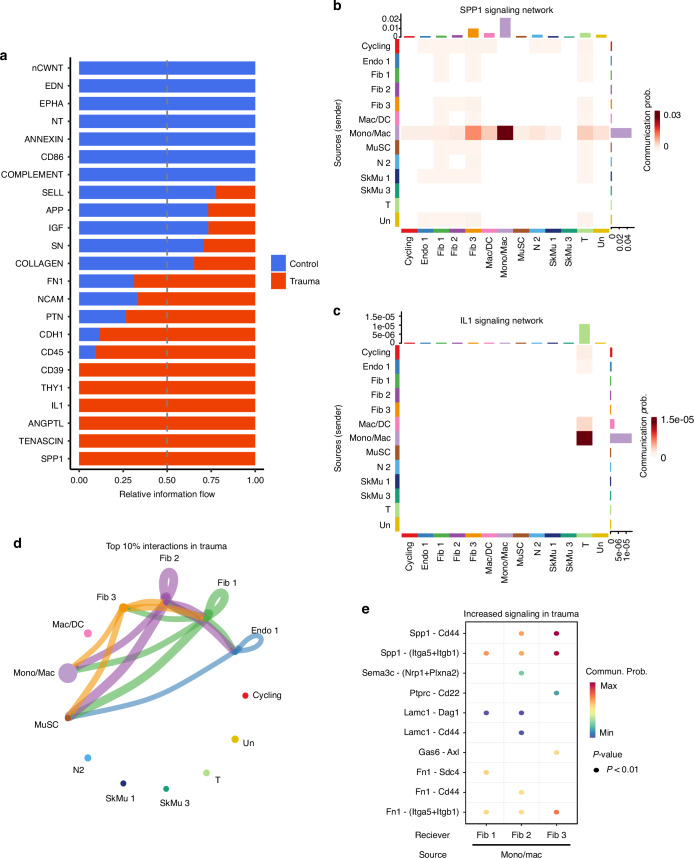
Mono/Mac cells drive inflammatory and fibrotic signaling via Spp1 networks in polytrauma. **a** Differential analysis identified key inflammatory pathways enriched in polytrauma, such as Spp1 and IL-1. Relative information flow, measured on a scale of 0 to 1, quantifies the signaling activity of each pathway, with higher values indicating greater activity. Mono/Mac clusters were shown to play prominent roles in the **b** Spp1 and **c** IL-1 signaling networks. **d** The top 10% of predicted cell-cell interactions, as determined by CellChat analysis, revealed increased interactions between Mono/Mac cells and fibroblasts. **e** Dot plot highlighting significant interactions upregulated in polytrauma, such as Spp1-Cd44, Spp1-Itga5+Itgb1, and Fn1-Itga5+Itgb1, from Mono/Mac cells to fibroblasts, as determined by CellChat

Next, we aimed to identify the signaling networks driving chronic inflammation in the local defect and the roles of Mono/Mac cells and other immune cells in these processes. Differential analysis revealed that inflammatory networks, including the *Spp1* and *Il-1* pathways, were highly enriched in polytrauma condition compared to control (Fig. 5a). Mono/Mac cells were identified as key mediators within these networks, utilizing these pathways to interact with both stromal and immune cells, such as fibroblasts and T cells (Fig. 5b, c). The predominant interactions in these networks were *Spp1-Cd44* and *Il18-(Il18R1+Il18rap)* (Fig. S12). Further analysis demonstrated that Mono/Mac cells predominantly communicated with fibroblasts in the polytrauma condition (Fig. 5d), emphasizing the importance of immune-stromal interactions in the post-polytrauma response. Notably, Mono/Mac cells exhibited increased interactions with fibroblasts compared to control, particularly through pathways linked to fibrosis, including *Spp1-Cd44*, *Spp1-Itga5+Itgb1*, and *Fn1-Itga5+Itgb1* (Fig. 5e).^30,31^ These findings highlight the pivotal role of Mono/Mac cell interactions, especially with stromal cells, in perpetuating chronic inflammation and fibrosis potentially contributing to impaired healing outcomes following polytrauma.

### Cross-tissue analysis of TIM and Mono/Mac cells

**Fig. 6:**
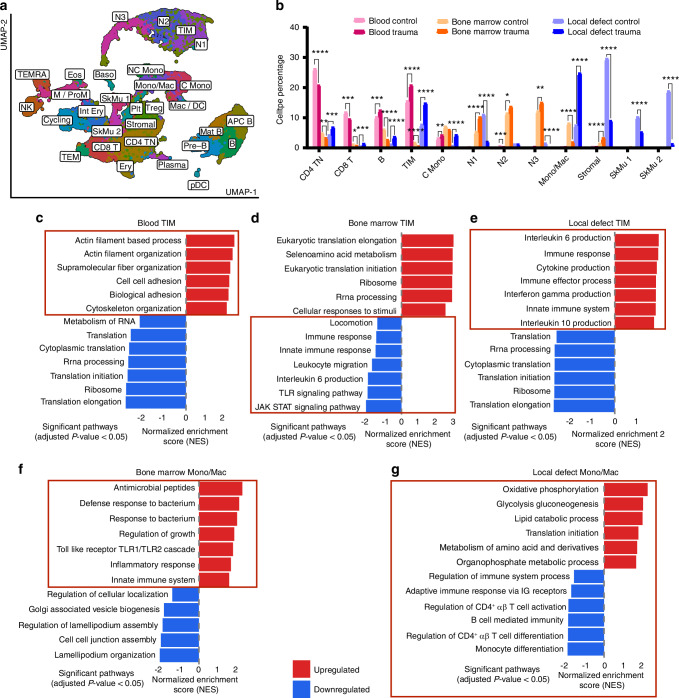
Cross-tissue analysis of TIM and Mono/Mac Cells reveal key biological differences across tissue compartments. **a** Integration and clustering of gene matrices from all tissues and conditions using uniform manifold approximation projection (UMAP). Clusters were identified using known marker gene expression, consistent with Fig. 2. **b** Cell type percentages across blood, bone marrow and local defect tissue in control and polytrauma conditions determine using all-tissue integrated dataset. *P*-values determined by chi-squared test of proportions. ****P < 0.000 1, *P < 0.05. Unmarked bars indicate non-significant differences. GSEA-identified global biological pathways differentially regulated in TIM cells (polytrauma vs. control) within (**c**) blood, (**d**) bone marrow and (**e**) local defect tissue. GSEA-identified global biological pathways differentially regulated in Mono/Mac cells (polytrauma vs. control) within (**f**) bone marrow and (**g**) local defect tissue. Red boxes highlight pathways discussed in the main text for TIM cells and Mono/Mac cells and their respective tissues. The Net Enrichment Score (NES) and *p*-value assessed the degree of gene set over-expression or under-expression in trauma compared to the control. A positive NES indicated upregulation, while a negative NES indicated downregulation

Following our identification of TIM and Mono/Mac cells as key mediators of polytrauma-induced immune dysregulation in the individual integrated tissue analysis, we conducted an integrated analysis combining all three tissues and conditions (henceforth, all-tissues integrated). This comprehensive approach allowed us to examine how the frequencies and biological responses, particularly of TIM and Mono/Mac cells, changed across tissue compartments following polytrauma. Consistent with our earlier findings, we confirmed the presence of major immune cell populations—including monocytes, neutrophils, T cells, B cells, and stromal cells—post-polytrauma (Fig. 6a).

To further investigate immune cell trafficking across tissue compartments following polytrauma, we analyzed the frequency of each cell population within each tissue (Fig. 6b). Our results revealed that T and B lymphocytes decreased in the bone marrow but increased in the local defect tissue in response to polytrauma. T cells also significantly decreased in the blood, which correlated with an expansion of TIM cells in the blood, characteristic of SIDIS. TIM cells also increased in the local defect tissue, underscoring their role in polytrauma-induced immune dysregulation, both systemically and locally. The frequency of Mono/Mac cells decreased in the bone marrow but increased substantially in the local defect tissue. As anticipated, local defect tissue-resident cells, such as skeletal muscle and stromal cells, were primarily found in the local defect tissue but decreased significantly in frequency following polytrauma. The trafficking patterns observed in this cross-tissue analysis were similar with the tissue-specific analysis. These findings highlight the dynamic and disrupted immune cell trafficking that contributes to polytrauma-induced immune dysregulation.

Given their elevated presence in the blood and local defect tissue, as well as their potential role in SIDIS, we investigated the broader biological responses of TIM cells across tissues following polytrauma. In the blood, TIM cells upregulated chemotaxis and migration pathways, including those involving cytoskeletal rearrangement and actin filament processes, suggesting movement between tissues (Fig. 6c). In the bone marrow, they downregulated key inflammatory pathways, including IL-6, Toll-like receptor, and JAK-STAT signaling, potentially contributing to SIDIS (Fig. 6d). Within the local defect tissue, TIM cells increased immune responses through interferon-gamma and IL-10 signaling, indicating activation at the injury site (Fig. 6e). This cross-tissue analysis reveals a progression from systemic immunosuppression to enhanced migration and localized activation, providing insight into how TIM cells may drive polytrauma-induced immune dysregulation.

Finally, we examined Mono/Mac cell responses to polytrauma across tissues because of their significant expansion and potential role in chronic inflammation. In bone marrow, Mono/Mac cells showed heightened inflammatory activation, marked by increased toll-like receptor 1/2 signaling and enhanced innate immune responses (Fig. 6f). However, in the local defect tissue these cells showed increased oxidative phosphorylation metabolism and suppressed T and B cell responses. This metabolic shift likely fuels their heightened inflammatory activity (Fig. 6g).^32,33^ Notably, these cells were absent in the blood samples. This distinct pattern—inflammatory activation in bone marrow and altered metabolism with suppression of adaptive immune responses at the injury site—suggests how Mono/Mac cells may drive chronic inflammation and impair healing after polytrauma.

### Global interaction analysis following polytrauma

**Fig. 7:**
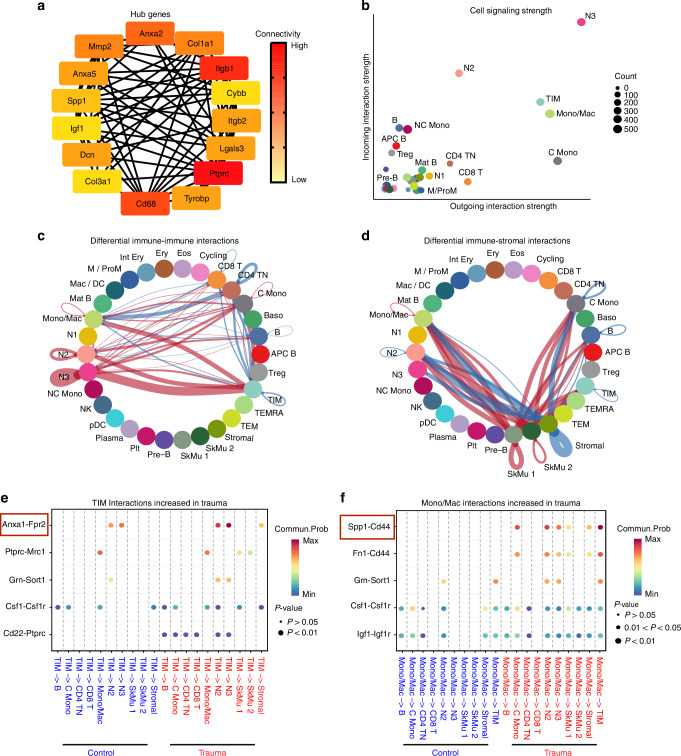
TIM and Mono/Mac cells mediate post-polytrauma communication via Anxa1-Fpr2 and Spp1-Cd44 pathways, respectively. **a** Top 15 hub genes from differentially expressed genes in polytrauma using all-tissue integrated dataset. **b** Dot plots visualize polytrauma-induced cell signaling strength. **c** Chord diagrams of differential immune cell interactions in polytrauma versus control condition. **d** Chord diagrams of differential interactions between immune and tissue-resident cells (red: upregulated, blue: downregulated in polytrauma). **e** Most significant ligand-receptor interactions with increased communication probability in polytrauma for TIM cell-initiated signaling. **f** Most significant ligand-receptor interactions with increased communication probability in polytrauma for Mono/Mac cell-initiated signaling

Using our all-tissue integrated dataset, we identified global patterns of immune dysregulation. These included key genes, immune cells, and cell-cell interactions that were dominant across all tissues rather than specific tissues. First, we identified polytrauma hub genes—highly connected genes that potentially act as key regulators of polytrauma-induced immune dysregulation. From the polytrauma dataset, we identified 15 hub genes, with *Ptprc (CD45), Itgb1*, and *Cd68* showing the highest connectivity (Fig. 7a). Notably, *Cd68*, a monocyte/macrophage marker, was significantly upregulated in Mono/Mac cells following polytrauma compared to controls. However, this upregulation was not observed in other monocyte or macrophage clusters (Fig. S13). In addition, Mono/Mac cells upregulated several other hub genes, including *Itgb2, Tyrobp, Lgals3*, and *Spp1* (Fig. S14a). Beyond Mono/Mac cells, hub genes were also regulated by TIM cells, N1 neutrophils, skeletal muscle cells (SkMu), and stromal cells (Fig. S14b). These findings identify genes with high connectivity in the post-polytrauma immune response, with Mono/Mac cells emerging as key regulators of hub genes.

Our analysis revealed heightened communication signaling by specific myeloid cell populations including neutrophil cluster 2 (N2), neutrophil cluster 3 (N3), classical monocytes (C Mono), TIM and Mono/Mac cells following polytrauma (Fig. 7b). N2 and N3 neutrophils displayed an energy-efficient activation profile, characterized by enhanced immune responses alongside reduced metabolic activity (Fig. S15). C Mono exhibited a stress-adaptive phenotype, upregulating survival pathways while compromising immune functions, particularly antigen presentation and adaptive immune responses (Fig. S16). TIM cells demonstrated increased chemotactic capacity while downregulating pro-inflammatory pathways (IL-17, TLR-9) (Fig. S17a). Upon closer inspection of TIM cells immune-related DEGs and pathways, IL-4/IL-13 and IL-10 pathways were upregulated while antigen presentation pathways were reduced (Fig. S17b, c). Lastly, Mono/Mac populations showed elevated metabolic processes with diminished adaptive immune responses (Fig. S18a). Upon closer inspection of Mono/Mac cells immune-related DEGs and pathways, these cells upregulated antigen-presentation and downregulated inflammation resolution pathways (IL-4/IL-13, IL-10) (Fig. S18b, c). TIM and Mono/Mac findings aligned with the individual integrated tissue analyses where TIM cells demonstrated an immunosuppressive phenotype and Mono/Mac cells were highly inflammatory. Overall, these results underscore the central role of myeloid cells, particularly neutrophils and monocytes/macrophages, in the polytrauma immune response.

Since TIM and Mono/Mac cells substantially increased in frequency, communication signaling, and regulated hub genes, we sought to investigate how their interactions with other immune cells differed in polytrauma compared to control. TIM cells increased communication with other myeloid cells especially Mono/Mac, N2 and N3, whereas interactions with and between adaptive immune cells such as Naive CD4 T Cells (CD4 TN), CD8 T and B cells decreased (Fig. 7c). Interestingly, interactions between immune cells and stromal cells decreased significantly after polytrauma but interaction with skeletal muscle cells increased (Fig. 7d). Consistent with their immunosuppressive phenotype, TIM cells significantly increased communication with other immune cells via immunosuppressive pathways *Anxa1-Fpr2* and *Ptprc-Mrc1* (Fig. 7e). These cells also significantly decreased several pathways that were used for communication between them and adaptive immune cells present in the control condition (Fig. S19a), further reinforcing their potential contribution to SIDIS through impaired antigen presentation and T cell responses. Meanwhile, Mono/Mac cells significantly increased communication with other immune cells via *Spp1-Cd44* and *Fn1-Cd44* pathways after polytrauma (Fig. 7f). These cells decreased communication using pathways such as *Gas6-Axl*, which are involved in inflammation resolution, supporting their role in perpetuate inflammation (Fig. S19b). These altered communication patterns reveal a global shift toward dominant myeloid cell interactions with concurrent suppression of adaptive immune responses and dysregulated immune-tissue-resident interactions, with TIM cells involved in SIDIS while Mono/Mac cells perpetuating inflammation.

## Discussion

Severe musculoskeletal trauma often leads to complications like nonunions, delayed healing, and infections despite advances in acute treatment.^1^ Our previous studies showed that circulating myeloid cells involved in SIDIS correlated negatively with bone healing outcomes, while adaptive immune cells correlated positively.^5,7^ These findings highlight the need for deeper characterization of immune cell populations following polytrauma to understand their roles in immune dysregulation and bone healing. Using single-cell RNA sequencing, we investigated immune cell phenotypes and molecular mechanisms to identify early predictive biomarkers of immune dysregulation. Individual integrated tissue analyses revealed two key cellular drivers. TIM cells in the blood expressed genes reflecting immunosuppression and downregulated effector functions such as antigen presentation, while Mono/Mac cells in local defect tissue upregulated proinflammatory responses and communication with fibroblasts via SPP1 signaling networks. Cross-tissue analyses and subsequent global interaction analyses, both performed using all-tissues integrated datasets (combining all tissues and conditions), demonstrated distinct biological responses of these cells across different compartments. The global interaction analysis revealed increased communication between TIM and Mono/Mac cells and other systemic and local immune cells through specific pathways. TIM cells increased communication via *Anxa1-Fpr2* and Mono/Mac cells through *Spp1-Cd44*. Together, our analyses support TIM and Mono/Mac cells as central mediators of polytrauma-induced immune dysregulation, with TIM cells driving immunosuppression and Mono/Mac cells promoting chronic inflammation. To the best of our knowledge, this is the first comprehensive characterization of immune cells in both systemic (blood and bone marrow) and local defect tissues that provides new insights into the molecular mechanisms underlying polytrauma-induced immune dysregulation.

We identified TIM cells as potential contributors of SIDIS. We have previously reported that CD11b^+^ HIS48^+^ myeloid cells in the blood expand following trauma, suppress T cells, and correlate negatively with bone healing in rats.^5,7^ These broad markers, however, identify both immunosuppressive and functional myeloid cells. Therefore, we performed transcriptomic analysis of blood myeloid cells expressing immunosuppressive genes associated with IL-4/IL-13, IL-10 or MDSCs to identify TIM cells and understand their molecular mechanisms in SIDIS. DEG and GSEA analysis on both individual integrated blood tissues and all-tissues integrated datasets confirmed TIM cells upregulate immunosuppressive genes and associated pathways while downregulating MHC-I genes and antigen-presentation pathways. One of the genes highly upregulated by these cells was *Anxa1*, which promotes M2 macrophage polarization and prevents cytotoxic T cell responses by inducing regulatory T lymphocytes.^34,35^ Both individual blood tissue analysis and global interaction analysis showed TIM cells increase communication through the *Anxa1-Fpr2* pathway, which is known to suppress innate and adaptive immune responses via NF-κB and MAPK pathways.^36^ While this pathway has been successfully targeted in cancer immunotherpay,^37,38^ its role in polytrauma requires further investigation to determine if pathway modulation could improve SIDIS. Further in vivo and in vitro experiments are needed to determine the specific role of *Anxa1* in SIDIS and broader immune dysregulation.

To identify therapeutically targetable TIM cells expanding after trauma, we performed flow cytometry analysis 5 days post-polytrauma. This analysis revealed that CD1d^+^CD11b^+^HIS48^+^ cells expanded both systemically in the blood and locally in defect tissue. CD1d, a member of the CD1 glycoprotein family, is broadly expressed on antigen-presenting cells and presents glycolipid antigens to NKT cells by engaging with their surface T cell receptors. Previous research has identified CD1d as a marker for monocytic MDSCs, which are known for their immunosuppressive activity in allogeneic stem cell transplant patients.^39^ Similarly, regulatory B cells utilize CD1d-mediated lipid presentation to activate iNKT cells, thereby downregulating T cell responses during excessive inflammation.^40^ These findings suggest that CD1d expression may mediate TIM cell immunosuppressive activity, particularly systemically. Furthermore, since CD1d^+^CD11b^+^HIS48^+^ cells represent a subset of CD11b^+^HIS48^+^ cells previously linked to poor bone healing, they are likely contributors to impaired healing outcomes. Therefore, CD1d serves as a valuable marker for identifying and isolating TIM cells both systemically and locally. Future studies will leverage this marker to further characterize TIM cells in vivo and investigate how modulating CD1d^+^ TIM cells impacts SIDIS and bone healing outcomes.

Mono/Mac cells were identified as key players in chronic inflammation, fibrosis, and impaired adaptive immune responses following polytrauma. Under normal conditions, these cells regulate inflammation, produce growth factors such as bone morphogenetic protein 2 (BMP-2) and platelet-derived growth factor (PDGF) and promote osteogenesis to support bone healing.^41^ However, our DEG analysis on both individual integrated local defect tissues and all-tissues integrated datasets revealed that Mono/Mac cells upregulate inflammatory genes (*Spp1, Cd68, Arg1*) and antigen presentation pathways, while downregulating inflammation resolution genes and pathways. Additionally, CellChat analysis showed enhanced upregulation of inflammatory *Spp1* and *Il-1* signaling networks post-polytrauma. Notably, *Spp1* encodes the multifunctional glycoprotein osteopontin, an important regulator of inflammation in various disease models and pathologies.^42,43^ These findings suggest that the sustained expression of pro-inflammatory factors by Mono/Mac cells potentially contributing to chronic and prolonged inflammation. Moreover, we observed Mono/Mac cells increased interactions with fibroblasts and upregulate genes *Spp1* (osteopontin), *Fn1* (fibronectin), and *Arg1* (Arginase 1). Macrophages expressing these genes were identified as profibrotic and have been involved in pulmonary, cardiac, and kidney fibrosis.^44,45^ While initial profibrotic activity facilitates tissue repair, prolonged extracellular matrix deposition can impair healing and result in nonunion.^14,46^ Both individual local defect tissue analysis and global interaction analysis revealed increased *Spp1-Cd44* signaling between Mono/Mac cells and myeloid, skeletal, and stromal cells following polytrauma. This pathway has been linked to dysregulated lipid metabolism and fibrosis in various pathologies.^47,48^ Although targeting this axis has shown promise in cancer treatment by restoring T cell responses and reducing tumor burden,^49^ its role in polytrauma requires further investigation through functional in vitro and in vivo studies. Finally, GSEA analysis revealed downregulation of T and B cell-related immune pathways, which are essential for fracture repair, as evidenced by their positive correlation with bone healing outcomes as seen in our previous studies.^7,50^ The lack of inflammation resolution has been implicated in precluding adaptive immune cells activation and moving towards a pro-repair phase. These findings identify Mono/Mac cells as potential therapeutic targets for addressing polytrauma-induced immune dysfunction, particularly in local defect tissues.

Cross-tissue and global interaction analysis at Day 5 post-polytrauma revealed widespread dysregulation of myeloid cell responses that likely impair bone healing. Cross-tissue analysis revealed significant expansion of myeloid populations, with increased neutrophils (i.e., N1, N2, N3) in the bone marrow and elevated C Mono, TIM, and Mono/Mac populations in blood and injured tissues. Global tissue interaction analysis showed increased signaling between these myeloid populations after polytrauma, indicating their active involvement in post-injury responses. While neutrophils and macrophages are known to be critical early mediators of fracture healing,^12^ our data revealed disrupted myeloid cell dynamics. Normally, neutrophils migrate to the fracture hematoma to stabilize the injury and recruit monocytes and macrophages.^12^ However, in our data, N2 and N3 cells expanded in the bone marrow but they did not increase at the injury site, suggesting impaired healing. TIM cells participated in SIDIS and activated both inflammatory and immunosuppressive pathways locally, demonstrating their role in fracture healing. The typical transition of monocytes and macrophages from inflammatory to immunosuppressive phase during fracture repair was significantly disrupted after polytrauma. Specifically, Mono/Mac cells exhibited elevated inflammatory pathways and oxidative metabolism while decreasing immunosuppressive responses, suggesting a shift toward persistent inflammation. Both C Mono and Mono/Mac populations exhibited downregulation of interactions with adaptive immune cells (T and B cells) and associated pathways, which are critical for fracture hematoma formation and balanced bone remodeling through osteoclastogenesis and osteoblastogenesis.^51^ This disrupted myeloid-adaptive immune cell communication likely impairs bone healing. Together, these findings demonstrate how polytrauma disrupts the crucial balance of immune cells, particularly myeloid responses essential for effective bone healing.

## Conclusion

Our study provides a comprehensive single-cell level understanding of both the systemic and local immune cell milieu and their functional relations following polytrauma. We identified TIM and Mono/Mac cells as pivotal drivers of polytrauma-induced immune dysregulation. TIM cells emerged as key mediators of SIDIS, promoting immunosuppression and impairing effector immune functions, such as antigen presentation, and were potentially involved in impaired bone healing outcomes. In contrast, Mono/Mac cells were identified as drivers of chronic inflammation and potential fibrosis via the Spp1 signaling network. These cells exhibited enhanced communication with other immune populations through *Anxa1-Fpr2* and *Spp1-Cd44* pathways, presenting potential molecular targets for therapeutic modulation. These insights not only deepen our understanding of immune interactions in response to polytrauma but also highlight actionable targets for therapeutic intervention. Future studies, including experimental validation, are essential to translate these findings into targeted strategies to modulate immune responses, reduce complications, and improve clinical outcomes in polytrauma patients.

## Materials and methods

### Polytrauma model

All animal experiments were performed in accordance with the Georgia Institute of Technology Institutional Animal Care and Use Committee (IACUC). As previously reported, unilateral 8 mm defects were created in the mid-diaphysis of the femur with an overlying 8 mm diameter full thickness quadriceps defect in 13-week-old Sprague Dawley rats (Charles River Laboratories) using an oscillating saw and biopsy punch.^4,5^ On Day 5 post-surgery, blood samples were obtained, along with bone marrow from the contralateral leg, and tissue samples were collected from the area adjacent to the defect site from each animal (*n* = 3). Blood, bone marrow, and local defect tissue was also collected from the naïve rats (*n* = 3) that did not have any injury. The animals were double-housed, maintained on a 12-h light/dark cycle, and allowed ad libitum access to food and water. On day 7 post-surgery, animals that had not been previously euthanized were euthanized by CO_2_ inhalation.

### Tissue collection and single-cell RNA-sequencing procedure

Blood was drawn via the rat tail artery and collected into 15 mL tubes. Red blood cells were lysed using 1X RBS lysis buffer (eBioscience). Blood was processed by lysing the red blood cells and the cell count and viability was determined. One million cells from each blood sample (*n* = 3) were collected and combined. The cells were fixed, resuspended in FACS buffer and stored at 4 °C. Bone marrow was obtained by dissecting the leg contralateral to the injury and isolating the femur. The cells were washed with PBS and 70% EtOH. The marrow cavity was exposed by cutting off the bone ends and marrow cells were flushed using a syringe containing PBS and 23-guage needle into a 50 mL tube. The cells were strained using a 40 μm cell strainer and red blood cells were lysed using 1x RBC lysis buffer. The cell viability was determined and 2 million cells from each bone marrow sample (*n* = 3) were combined into a final collection tube. The cells were stored in FACS buffer at 4 °C prior to processing. Lastly, the tissue adjacent to the defect site was cut and incubated for ~30 min in 2 mg/mL of collagenase D in optiMEM. The cell suspension was strained through 40 um cell strainer and the red blood cells were lysed. The cell viability and count were determined and 2 million cells from each defect sample (*n* = 3) were combined and collected. Cells were stored in FACS buffer at 4 °C. Cell number and viability was confirmed to achieve the target 5 000 barcoded cells for each tissue. Single-cell RNA sequencing (scRNAseq) was performed using 10x Genomics Chromium v3.1 kit (Cat. No. 1000121) to generate single-cell transcripts, according to the manufacturer’s instructions (protocol rev C). Libraries were sequenced on Nextseq500 (Illumina) and data were de-multiplexed, aligned and counted using Cell Ranger version 3.1.0.

### Single-cell RNA sequencing data preprocessing

Tissue samples from rats from different conditions (no injury, trauma, and treatment) were collectively analyzed across various tissue types (blood, bone marrow, and local defect tissue). Doublet scores were computed for each cell in every tissue sample using the Scrublet package in Python v3.^52^ Blood cells exceeding ±3 times the mean absolute deviation of total RNA counts, along with those with a mitochondrial DNA percentage exceeding 15%, were excluded. Subsequent processing utilized Seurat v4.2.2 to eliminate cells possessing fewer than 500 total RNA counts and more than 0.3% globin genes.^53^ Regarding bone marrow cells, the criterion for percent globin genes was not applied, while for cells from the local defect tissue, those with a mitochondrial DNA percentage exceeding 20% were excluded. Normalization of gene expression matrices for each tissue sample was performed using the SCTransform method, with mitochondrial counts regressed out.^54^ Cell cycle scores for S and G2/M phases were determined using mouse homologs gene list.^55^ To identify cells, SCT-normalized transcript counts were combined with Seurat using the top 4 000 variable genes, with the top 3 000 variable genes serving as anchors.^56^ Additionally, the influences of cell cycle scores were eliminated. Clustering of cells was conducted initially based on RNA integrated matrices on a per-tissue basis (e.g., all blood samples were integrated together, as were all bone marrow samples, etc.). Identifying clusters using marker genes and subclustering of metaclusters (e.g., T, B, macrophage/monocytes) were performed to identify and remove small doublet clusters co-expressing canonical markers of multiple cell types. Additionally, clusters expressing combinations of marker genes from multiple lineages or showing a high proportion of mitochondrial or cycling genes as markers were filtered out. The analysis resulted in 10 482 cells and 13 058 unique genes in blood, 7 136 cells and 13 493 unique genes in bone marrow and 5 443 cells and 15 058 genes in local defect tissue including cells from control and trauma groups. Once these cells were defined, they were analogously re-integrated, clustered, and annotated on a full tissue integration basis, such that conserved and unique cell types from the blood, bone marrow, and local defect tissue across all treatments could be identified. This processing resulted in four Seurat objects from which further downstream biological analysis was performed: a blood object, a bone marrow object, a local defect object, and a full tissue object (including all 23 061 cells).

### Cell type annotation, differential gene expression (DEG), Reactome pathway over-representation analysis (ORA), and gene set enrichment analysis (GSEA)

Cluster marker genes were calculated using Seurat by conducting a Wilcoxon Rank Sum (WRS) test on SCTransformed transcripts for each integrated cluster against all other clusters. Cell types in each Seurat object were assigned based on marker genes reported in the literature.^16,18,20–22,57,58^ A comprehensive list of cell type markers provided in Supplementary Excel File. Differentially expressed genes (DEGs) comparing the trauma group to the control group across all cell types were identified using a WRS test. Supplementary data containing DEGs from trauma groups compared to the control groups for blood, bone marrow, local defect tissue, and the full tissue integration are available in Supplementary Excel File. For TIM cells, DEGs were similarly determined by comparing gene expression between these cells and other trauma immune cells.

The Reactome pathway over-representation analysis (ORA) was conducted using significant DEGs to determine which immune system pathways were over-represented in the given list of DEGs. The Reactome database was selected for its thorough hierarchical annotation of numerous immune-related pathways. To understand the broader impact of trauma on the gene expression profile across all immune cells in each tissue type, ORA was performed on the top 200 most significant upregulated and downregulated DEGs. The selected genes were submitted to the Reactome database. Before analysis was performed, all non-human genes were mapped to their human equivalents. Significance criteria was set as *P*-value < 0.01 and false discovery rate (FDR) < 0.05. As reported in Kim et al., pathways with *P*-value < 0.01 and FDR < 0.15 were also reported and denoted the confidence of ‘possible’ or ‘hypothesis’.^60^ Additionally, ORA was conducted on the genes of each myeloid subcluster to identify clusters associated with immunosuppressive pathways using top 10–200 most significant genes from the list. Gene set size was variable given that some subclusters had fewer genes compared to others. The select genes were submitted to the Reactome database, the genes identifiers were converted to human orthologs and pathways enriched in the gene list were determined. Significance criteria was set as *P*-value < 0.01 and false discovery rate (FDR) < 0.05.

Gene set enrichment analysis (GSEA) was performed on DEGs expressed in at least 5% of cells for all cell types comparing trauma vs. control across all tissues using the fgsea package in R with a minimum bin size of 15 and no maximum with DEGs arranged according to log_2_(fold change). Rat gene lists from Hallmark sets, KEGG pathways, Gene Ontology Biological Process (GOBP), and Reactome were used for GSEA as provided by the msigdbr package using the Molecular Signature Database v7.4.^61^ The results of significant GSEA enrichments for the trauma group vs. the control comparisons for each tissue and the full tissue integration are provided in Supplementary Excel File.

### CD11b^+^HIS48^+^ cell sorting and processing for single-cell RNA sequencing

Blood was drawn via the rat tail artery and collected into tubes. Red blood cells were lysed using 1X RBS lysis buffer (eBioscience) and the cell count and viability was determined. The blood was processed and analyzed using flow cytometry. Based on previous finding, flow cytometry revealed two distinct subpopulations of CD11b,^+^ HIS48^+^ cells, a high side scatter population and the low side scatter population. Both the high and low side-scatter sub-populations were sorted and sequenced independently. Single-cell RNA sequencing (scRNA-seq) was performed using 10x Genomics Chromium v3.1 kit (Cat. No. 1000121) to generate single-cell transcripts, according to the manufacturer’s instructions (protocol rev C). Libraries were sequenced on Nextseq500 (Illumina) and data were de-multiplexed, aligned and counted using Cell Ranger version 3.1.0. For both the CD11b,^+^ HIS48^+^ sub-population samples, quality control, cell-cycle gene regression and normalization were performed as detailed in the section single-cell RNA sequencing data preprocessing. To identify trauma immune cell clusters with transcriptomic similarities to the CD11b,^+^ HIS48^+^ sub-populations, the CD11b,^+^ HIS48^+^ samples were independently integrated with blood trauma and treatment samples, as well as with bone marrow trauma and therapeutic treatment samples. Subsequently, clusters were identified and annotated. Downstream analysis was performed on the integrated datasets to identify immunosuppressive myeloid clusters.

### TIM Characterization in vivo studies

As previously reported, unilateral 8 mm defects were created in the mid-diaphysis of the femur with an overlying 8 mm diameter full thickness quadriceps defect in 13-week-old Sprague Dawley rats (Charles River Laboratories) using an oscillating saw and biopsy punch.^4,5^ The rats were double-housed with ad libitum food and water access on a 12 h light/dark cycle. On Day 5 post-surgery, blood samples were collected from the tail artery of naïve (*n* = 6) and polytrauma (*n* = 6) rats using Vacuette needles into lithium-heparin Microtainer tubes, followed by euthanasia. Blood samples underwent RBC lysis using 1X buffer (eBioscience), while local defect tissues were processed using Miltenyi skeletal muscle dissociation protocol. For flow cytometry analysis, cells from both blood and local defect tissue samples were blocked with anti-rat CD32, stained with UV Zombie live/dead stain, and labeled with antibodies against CD11b, HIS48, CD80, CD1d, and CD44. After fixation, cells were resuspended in FACS buffer (0.5% BSA in PBS) and analyzed using a Cytek Aurora 5 L Spectral Cytometer with FlowJo software, with gates established using FMO controls.

### Hub genes

Using the all-tissue integrated dataset, we identified the top 200 most significant differentially expressed genes (DEGs) between polytrauma and control conditions (adjusted *P*-value < 0.05, log_2_FC > 0.25 or log_2_FC < −0.25), excluding ribosomal and mitochondrial genes. These genes were analyzed using STRING database (v11.5, Rattus norvegicus) to generate a protein-protein interaction network, considering source evidence with a minimum combined score threshold of 0.4. Hub gene analysis using the Cytoscape CytoHubba plugin Degree and Shortest Path ranking methods revealed the 15 most influential genes within this network.

### CellChat

Clusters with fewer than 10 cells in at least one treatment were excluded from communication analysis, as defined by Seurat. Rat genes were converted to their mouse homologs, given that the CellChat database currently supports mouse and human genomes. Using transcript matrices for each treatment group, probable cell-cell interaction networks were computed with the CellChat package in R 4.2.2, utilizing predefined pathways such as “Secreted Signaling,” “ECM Receptor,” and “Cell-Cell Contact.“^59^ Default settings were employed for unsorted single cells. Statistically significant interactions were visualized through heatmap, circle plots, and stacked barplots, while gene expression was illustrated using violin plots.

## Supplementary information

Supplementary document

Revised DEG and GSEA Raw Data Files

## Data Availability

Clustering and visualization, DEG analysis, over-representation analysis, GSEA and CellChat data are available within the article and supplementary materials. All raw data generated or analyzed during this study are available from the corresponding authors on reasonable request.
