# Supplementary material for: Single-cell transcriptomic analysis identifies systemic immunosuppressive myeloid cells and local monocytes/macrophages as key regulators in polytrauma-induced immune dysregulation
Source: Bone Res. 2025 Jul 7;13:69. doi: 10.1038/s41413-025-00444-x (PMC12234669; doi:10.1038/s41413-025-00444-x)
Supplement: Supplementary file 1 — Supplementary document [file 41413_2025_444_MOESM1_ESM.docx]

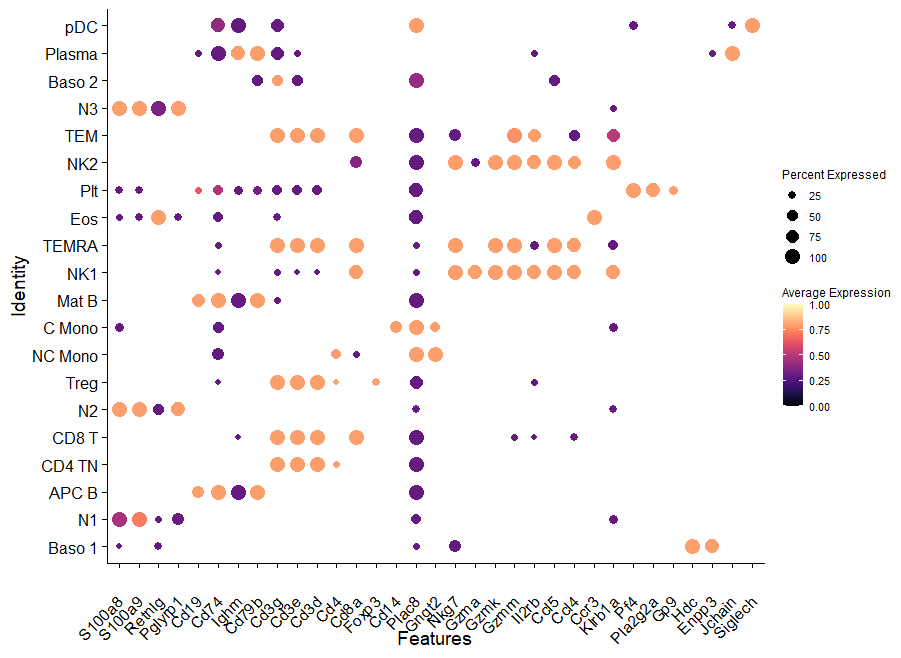

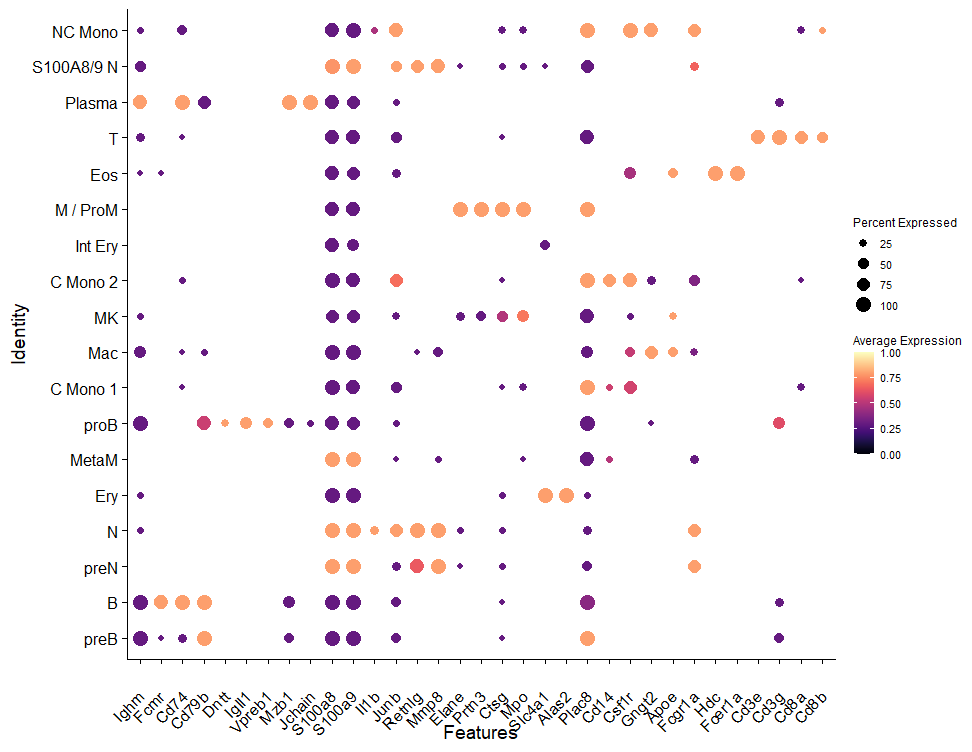


**a**

**b**

**Figure S1. Related to Figure 2.** Marker genes for identification of cell types present in the blood and bone marrow. Dot plots showing the percent and average expression of marker genes for the identification of heterogenous cell populations in the (a) blood and (b) bone marrow.


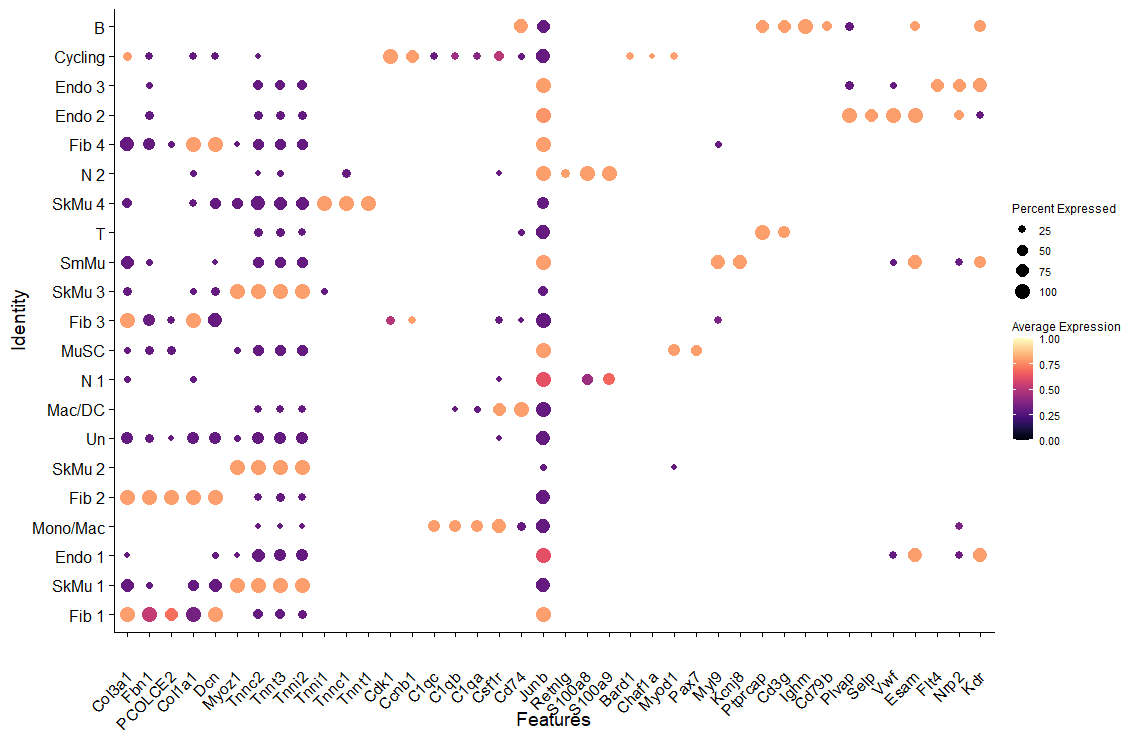


**Figure S2. Related to Figure 2.** Marker genes for identification of cell types present in the local defect tissue. Dot plots showing the percent and average expression of marker genes for the identification of heterogenous cell populations in the local defect tissue.

|  | **Cell Type** | **Marker Genes** |
| --- | --- | --- |
| **Myeloid** | Neutrophils | *S100a8, S100a9, Retnlg, Pglyrp1, Mmp8, Junb* |
|  | Pre-Neutrophils | *Ngp, Camp* |
|  | Metamyelocytes | *Np4, RatNP-3b, Camp, Mpo* |
|  | Myelocyte/Promyelocyte | *Elane, Prtn3, Ctsg, Mpo, Rnase2* |
|  | Eosinophils | *Ccr3, Retnlg, Fcer1a* |
|  | Basophils | *Hdc, Enpp3* |
|  | Classical Monocytes | *Cd14, Plac8, Vim* |
|  | Non-Classical Monocytes | *Gngt2, Plac8, Csf1r* |
|  | Macrophages | *Apoe, Fcgr1am Gngt2, Csf1r, C1q* |
|  | Plasmacytoid Dendritic Cells | *Siglech* |
| **Lymphocytes** |  |  |
|  | T Cells | *Cd3e, Cd3g, Cd3d* |
|  | CD4 T cells | *Lef1, Cd40lg* |
|  | CD8 T cells | *Cd8a* |
|  | Regulatory T Cells | *Foxp3, Icos* |
|  | CD8 Effector Memory T Cells with CD45RA (TEMRA) | *Cd8a, Ccl5, Ccl4, Gzmm, Gzmk, Nkg7* |
|  | CD8 Effector Memory T Cells (TEM) | *Cd8a, Cd3e, Cxcr3* |
|  | Natural Killer (NK) Cells | *Il2rb, Nkg7, Gzma, Gzmk, Klrd1, Klrb1a* |
|  | Antigen Presenting B Cells | *Cd74, Ebf1, RT1-genes* |
|  | Mature B Cells | *Ighm, Cd79b* |
|  | Fibroblasts | *Col3a1, Fbn1, Dcn, Col1a1* |
|  | Skeletal Muscle Cells | *Myoz1, Tnn* |
|  | Smooth Muscle Cells | *Myl9, Kcnj8* |
|  | Endothelial Cells | *Epas1, Kdr, Plvap, Selp, Vwf, Esam, Flt4, Nrp2* |
|  | Muscle Stem Cells | *Myod1, Pax7* |

**Figure S3. Related to Figure 2 and Figure 6.** Summary of marker genes for identification of all cell types across tissues.


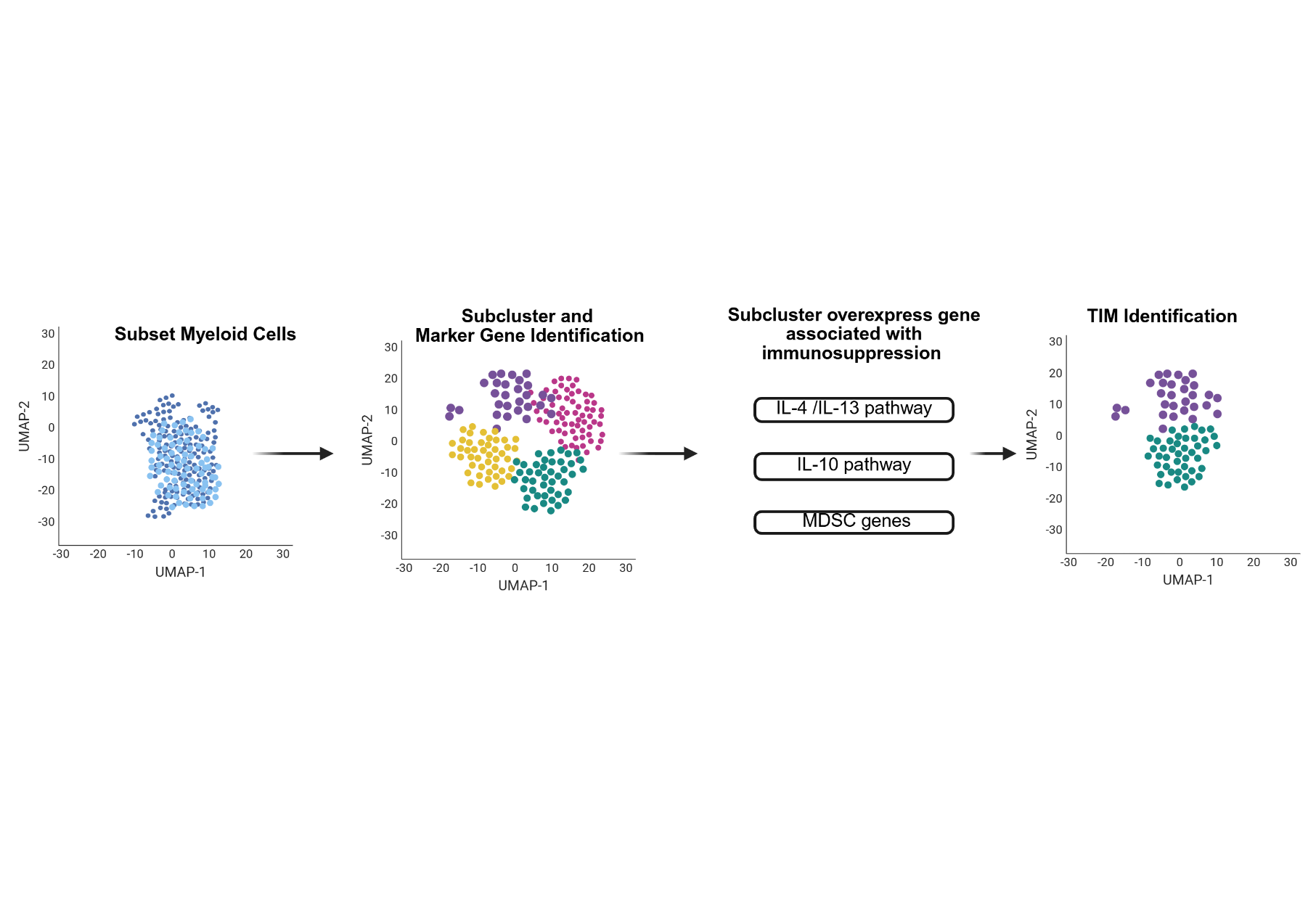


**Figure S4. Related to Figure 3.** Schematic representation of blood TIM identification process which 269 isolated myeloid cells and assessed their expression for genes linked to immunosuppressive pathways such as IL-4/IL-13, IL-10, or those associated with myeloid-derived suppressor cells.


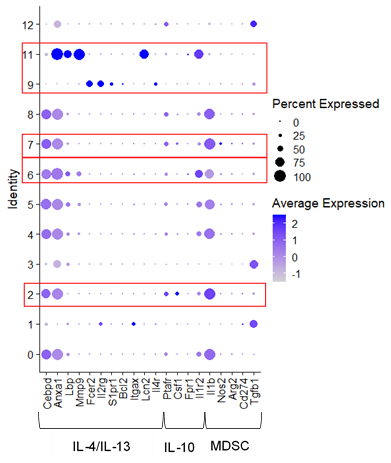


**b**


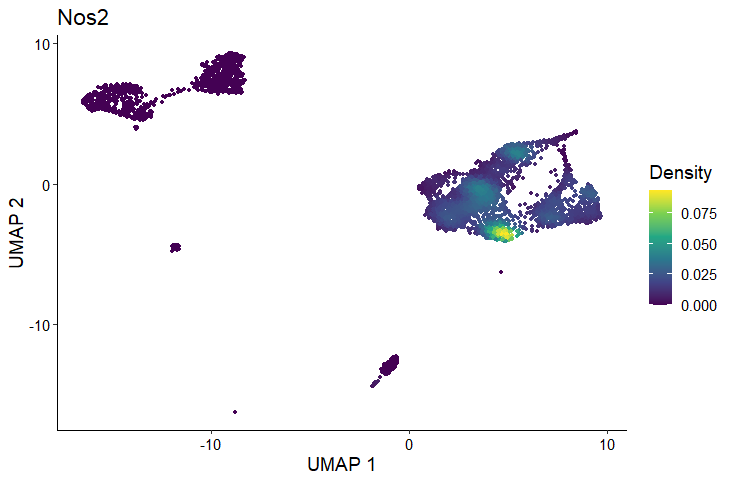

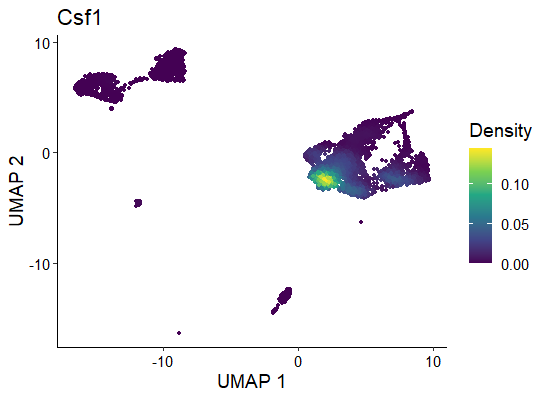

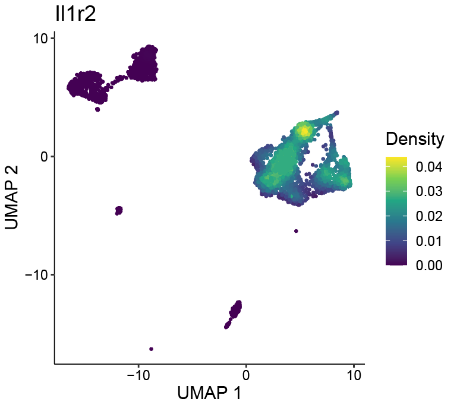


High Expression Cluster 2

High Expression Cluster 6

High Expression Cluster 7

**c**

**a**


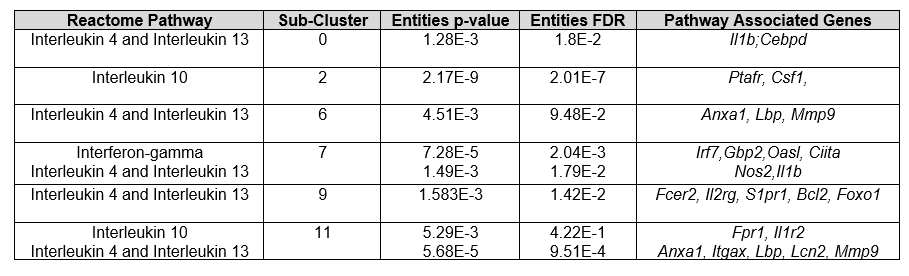


**Figure S5. Related to Figure 3.** Identification of immunosuppressive subclusters in the blood based on the expression of genes associated with immunosuppressive pathways, including IL-4/IL-13, IL-10, or myeloid-derived suppressor cells (MDSCs). (a) Marker genes were identified for each subcluster, revealing subsets expressing genes linked to immunosuppressive pathways, such as IL-4/IL-13 and IL-10. Significant pathways were identified using Reactome over-representation analysis (ORA). (b) Dot plot illustrating the expression of individual immunosuppressive genes across subclusters. (c) Density plot showing subclusters expressing ***Csf1, Il1r2,*** and ***Nos2.***

**Myeloid-derived suppressor genes**

**Joint density expression predominant clusters 7**


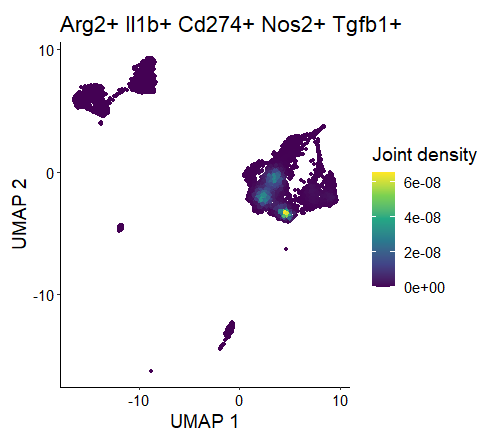


**IL-10 signaling genes**

**Joint density expression predominant clusters 2**


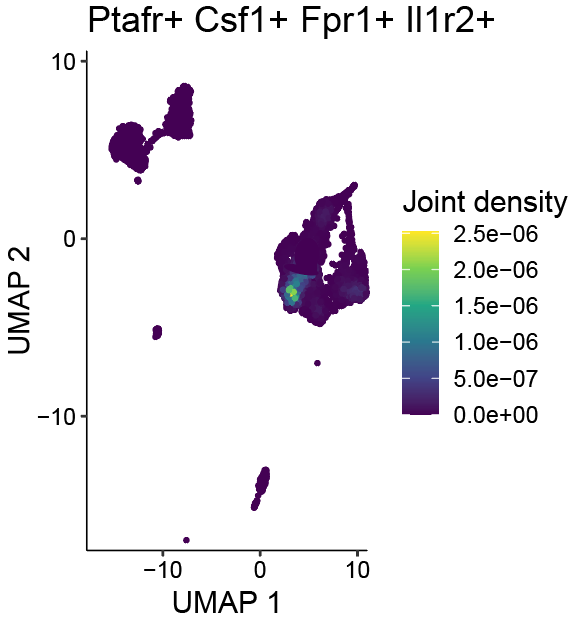


**IL-4/IL-13 signaling genes**

**Joint density expression predominant clusters 2 and 6**


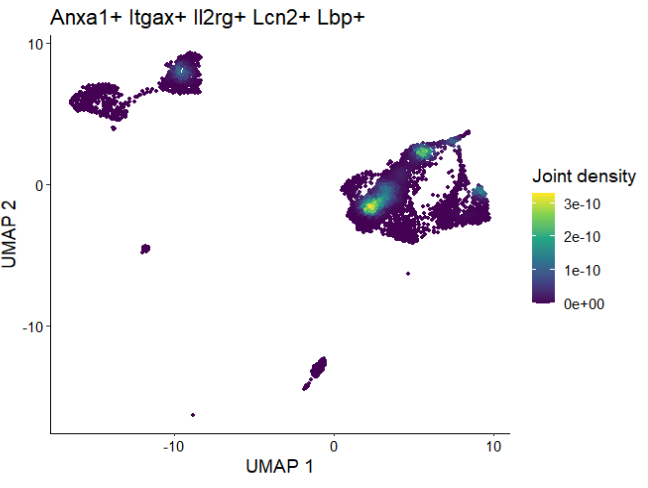


**a**

**b**

**c**

**Figure S6. Related to Figure 3.** Joint density plots showing the expression of multiple genes associated with (a) IL-4/IL-13, (b) IL-10, and (c) myeloid-derived suppressor cell pathways to identify trauma-induced immunosuppressive myeloid cells.


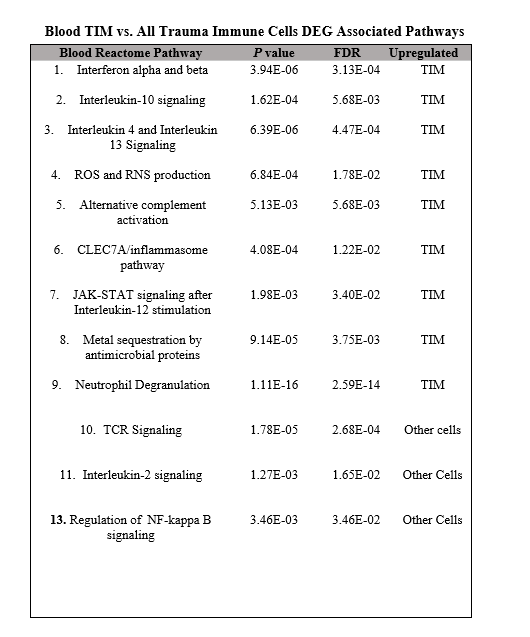


**Pathways Associated with Differentially Expressed Genes in Blood TIMs Compared to Other Trauma Immune Cells**

**Figure S7. Related to Figure 3.** Pathways upregulated by blood TIM cells compared to other cells in the polytrauma microenviorment. Over representation analysis (ORA) was conducted on the top 200 most up- and downregulated DEGs, and the significant immune system pathways were determined for. The significance cutoff for the pathways was set as p-value < 0.01 and false discovery rate (FDR) < 0.05.

**a**


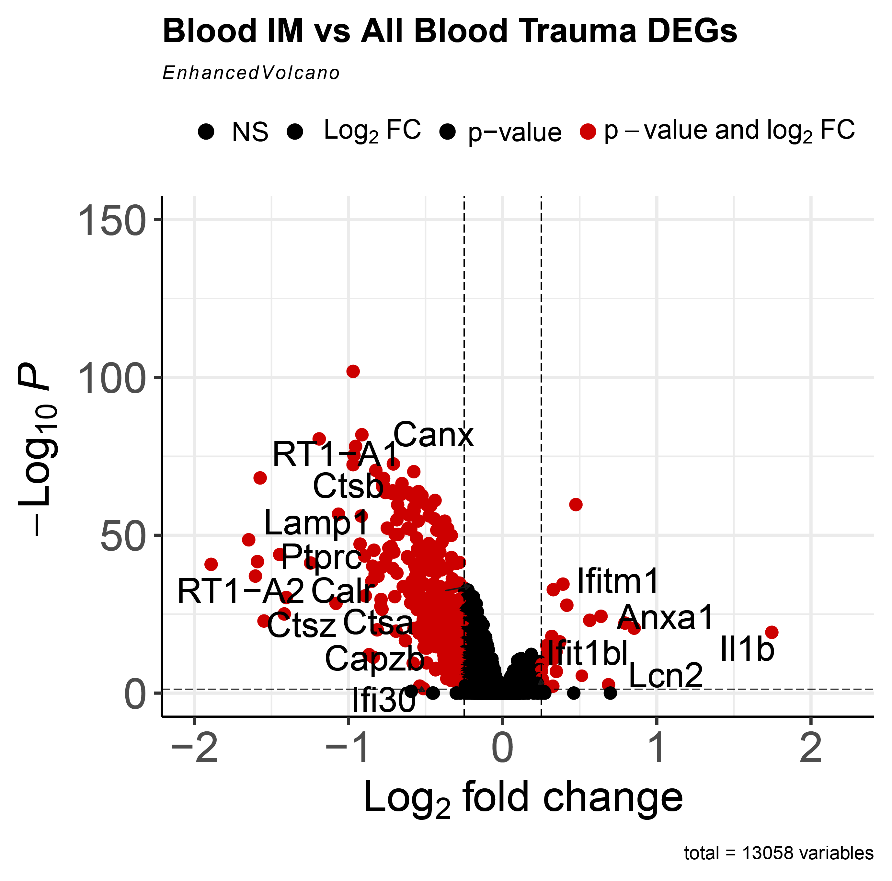


**Blood TIM vs Control Myeloid Cells**


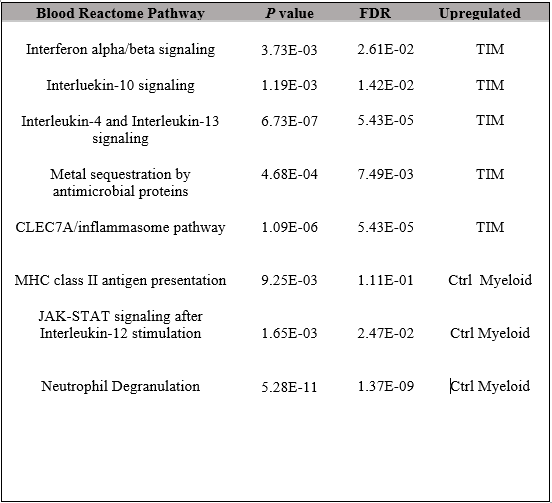


**b**

**Blood TIM vs. Control Myeloid Cells DEG Associated Pathways**

**Figure S8. Related to Figure 3.** Differentially expressed genes (DEGs) and their associated immune pathways identified from comparing polytrauma TIM cells to control myeloid cells. (a) DEG using Wilcoxon Rank Sum test were identified with the cutoff set as p-value adjusted < 0.05 and log-fold change > 0.25 for upregulated genes and < -0.25 for downregulated genes. The –Log_10_ (p values) indicates the level of significance of each gene while Log_2_ fold change represents the difference between the levels of expression for each gene between the trauma and control groups (baseline). (b) Over representation analysis (ORA) was conducted on the top 200 most up- and downregulated DEGs, and the significant immune system pathways were determined. The significance cutoff for the pathways was set as p-value < 0.01 and false discovery rate (FDR) < 0.05.


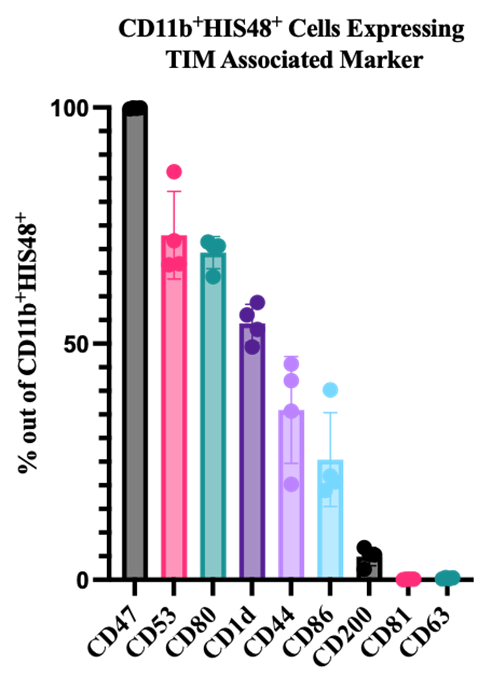

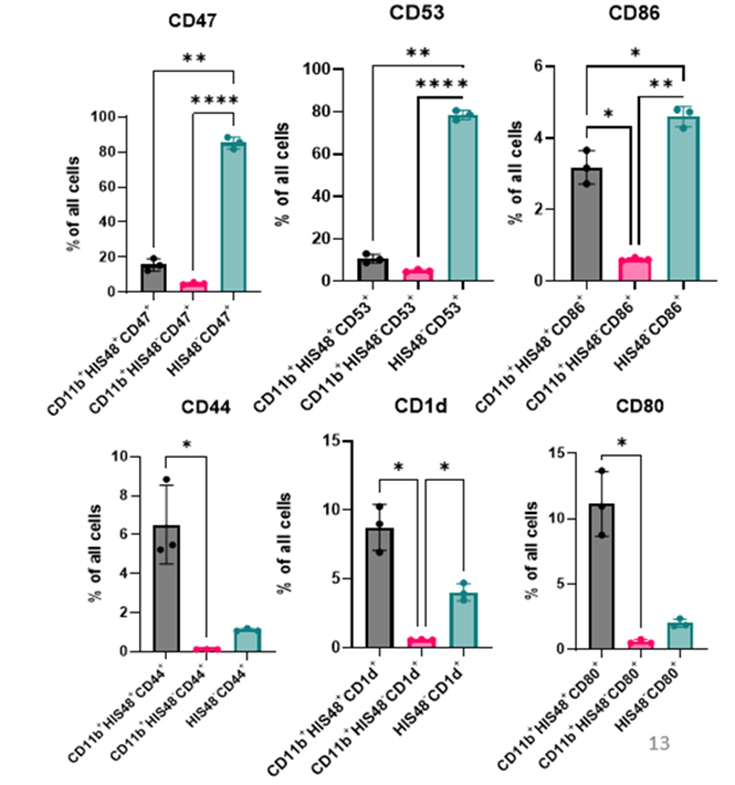


**% of live cells**

**% of live cells**

**% of live cells**

**% of live cells**

**% of live cells**

**% of live cells**

**a**

**b**

**Figure S9. Related to Figure 3.** Cluster of differentiation (CD) genes expressed by TIM cells. (a) Blood CD11b^+^HIS48^+^ cells isolated from polytrauma rats at Day 4 following injury expressed TIM markers CD47, CD53, CD80, CD1d, CD44 and CD86. (b) TIM markers CD47, CD53 and CD86 showed higher expression on HIS48- cells compared to CD11b^+^HIS48^+^ cells, while CD44, CD1d and CD80 showed higher expression on CD11b^+^HIS48^+^ cells compared to HIS48^-^ cells. Significance was determined using repeated measures one-way ANOVA with Tukey's post-hoc comparison or non-parametric Friedman test with Dunn's post-hoc comparison. Assay replicate n=3, ****P < 0.0001, ***P < 0.001, **P < 0.01, *P < 0.05


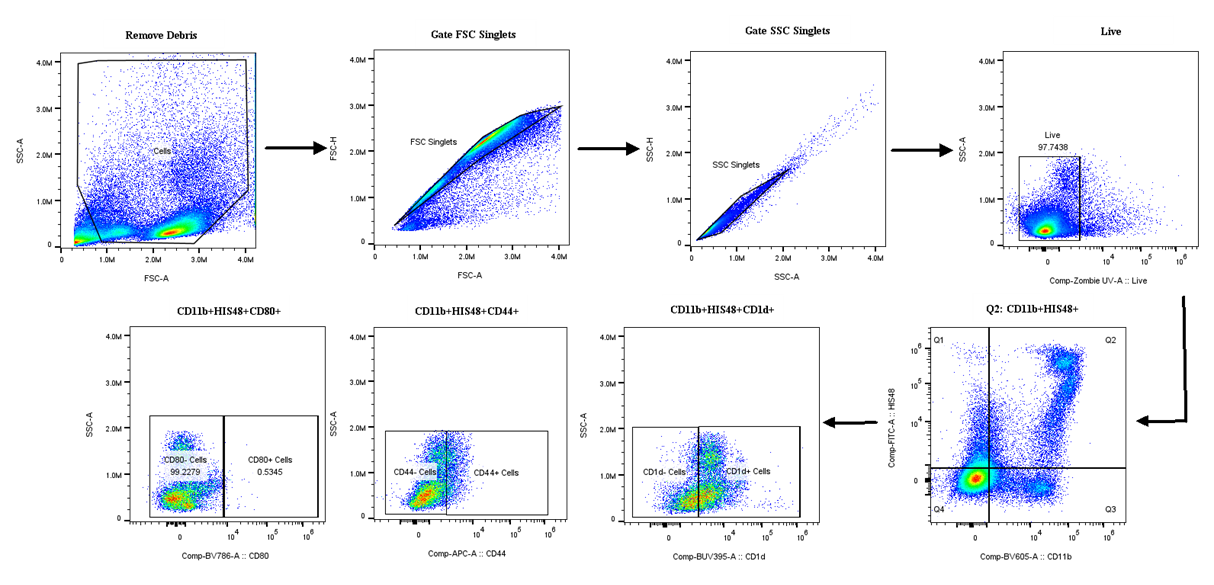


**Figure S10. Related to Figure 3.** Flow cytometry gating strategy for identifying CD11b^+^HIS48^+^ cells and TIM subset within CD11b^+^HIS48^+^ populations in both the blood and local defect tissue.


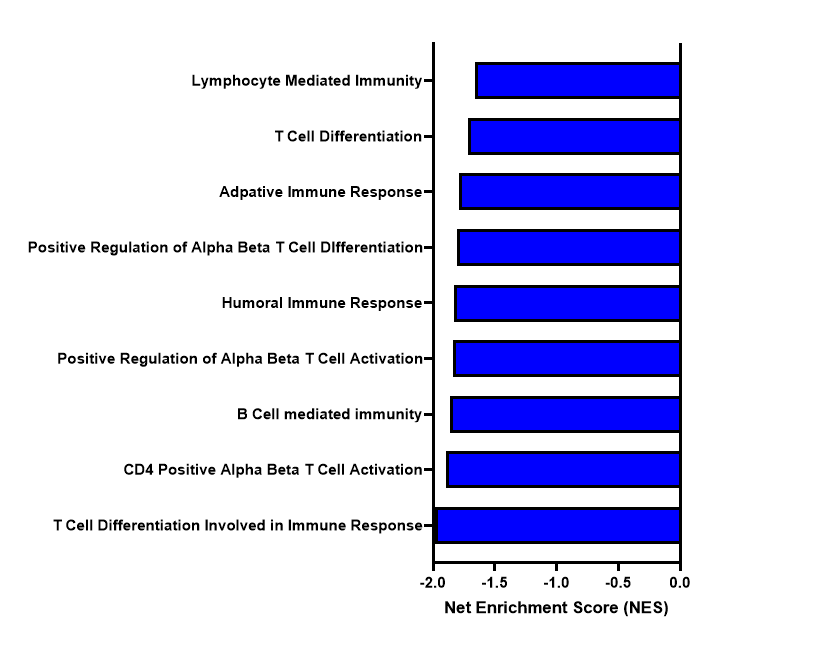


Significant Pathways

Adjusted *p-value* < 0.05

Downregulated in

Trauma

Upregulated in

Trauma

**Figure S11. Related to Figure 4.** Barplot of adaptive immune pathways downregulated in Mono/Mac cells identified by GSEA analysis. The Normalized Enrichment Score (NES) and p-value assessed the degree of gene set over-expression or under-expression in trauma compared to control. A positive NES indicated upregulation, while a negative NES indicated downregulation.


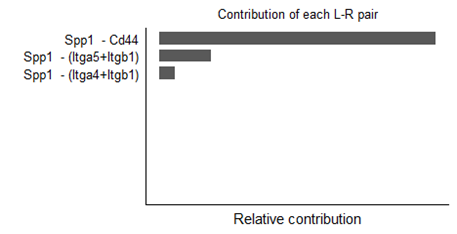

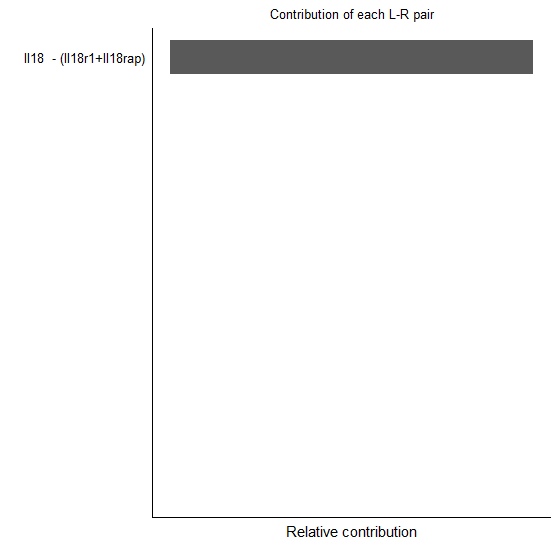


**a**

**b**

**Figure S12. Related to Figure 5.** Mono/Mac cells involved in Spp1 and Il-1 signaling network pathways increased post-polytrauma in local tissue. (a) Barplot showing *Spp1-Cd44* ligand-receptor interaction as the predominant pathway in the Spp1 network. (b) Barplot showing *Il18-(Il18r1-Il18rap)* ligand-receptor interaction as the predominant pathway in the Il-1 network.


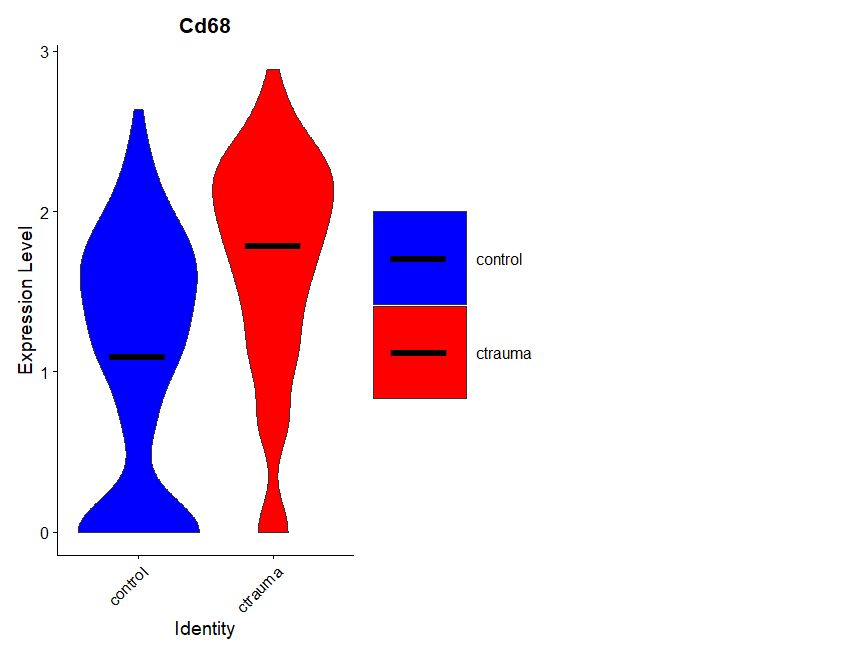


**Mono/Mac**

**Mac/DCs**


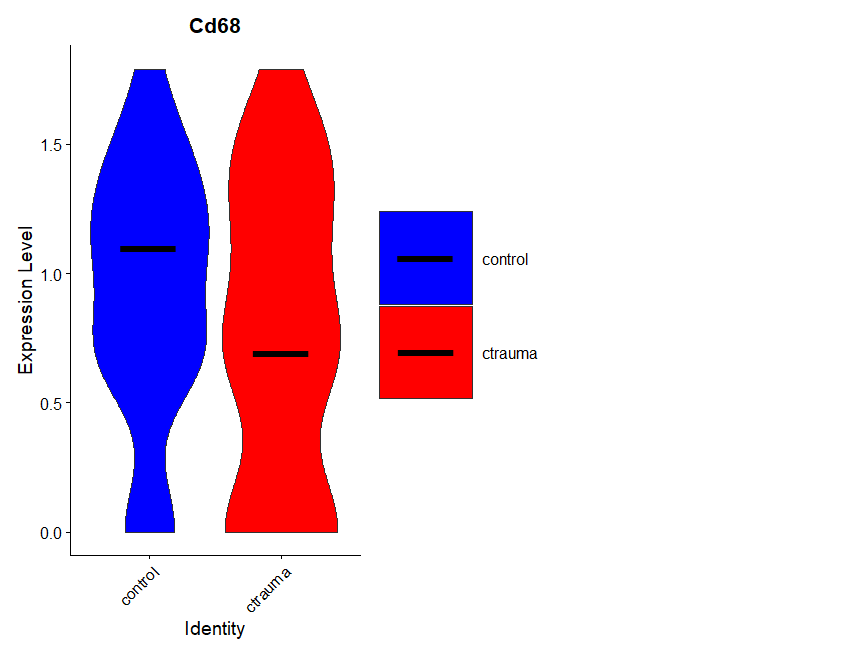

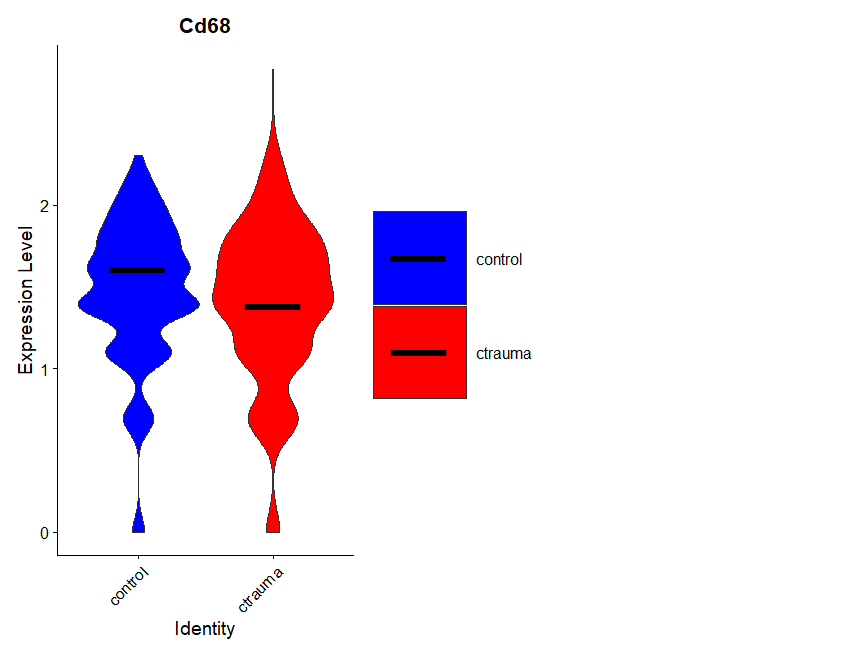


**C Mono**


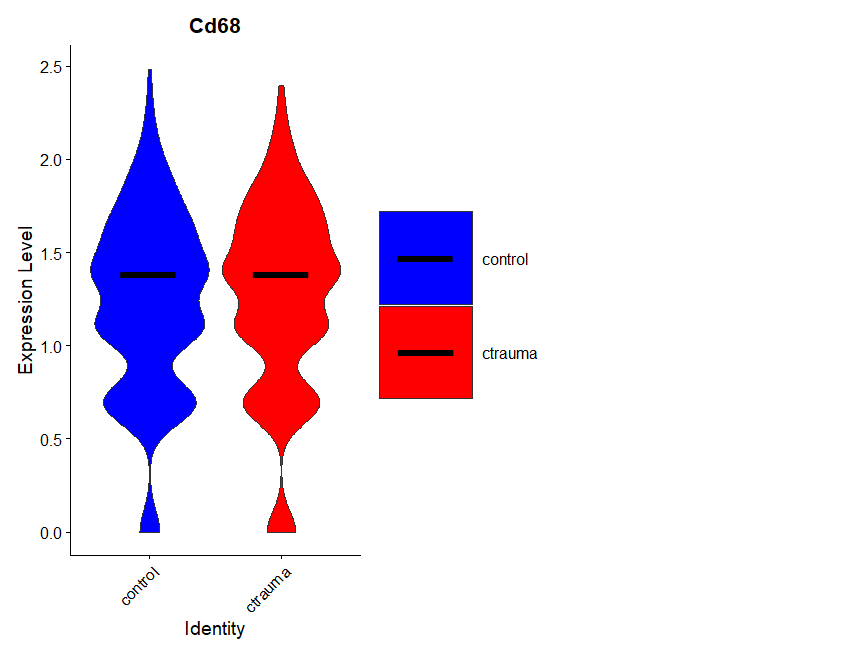


**NC Mono**

**Figure S13. Related to Figure 7.** Violin plot showing Cd68 expression distribution across monocyte and macrophage clusters, with median expression indicated by center line.


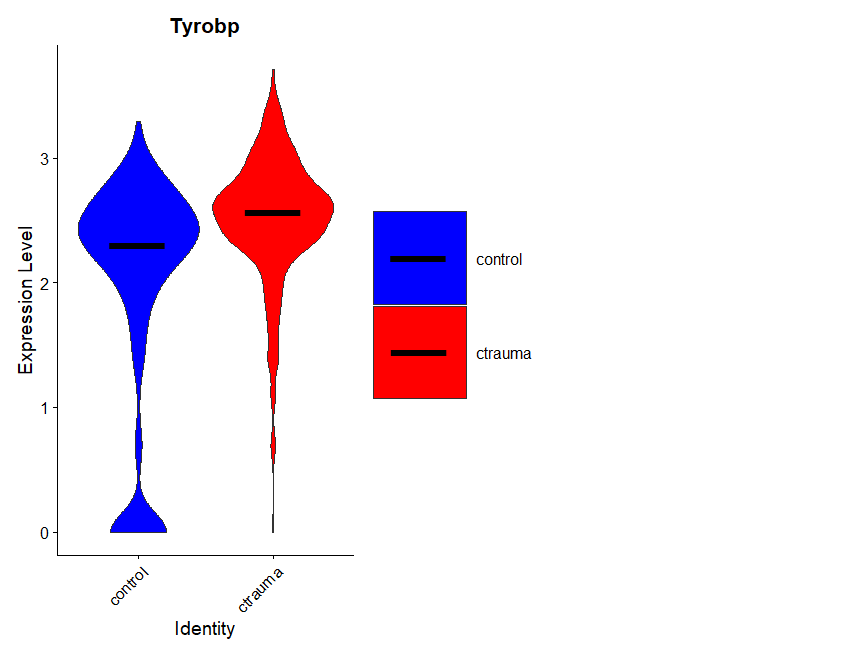

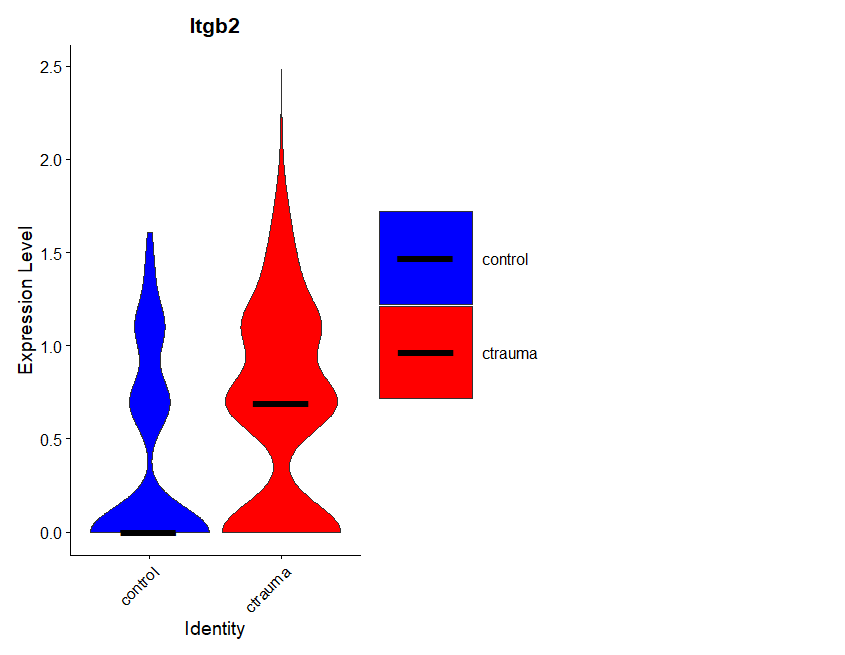

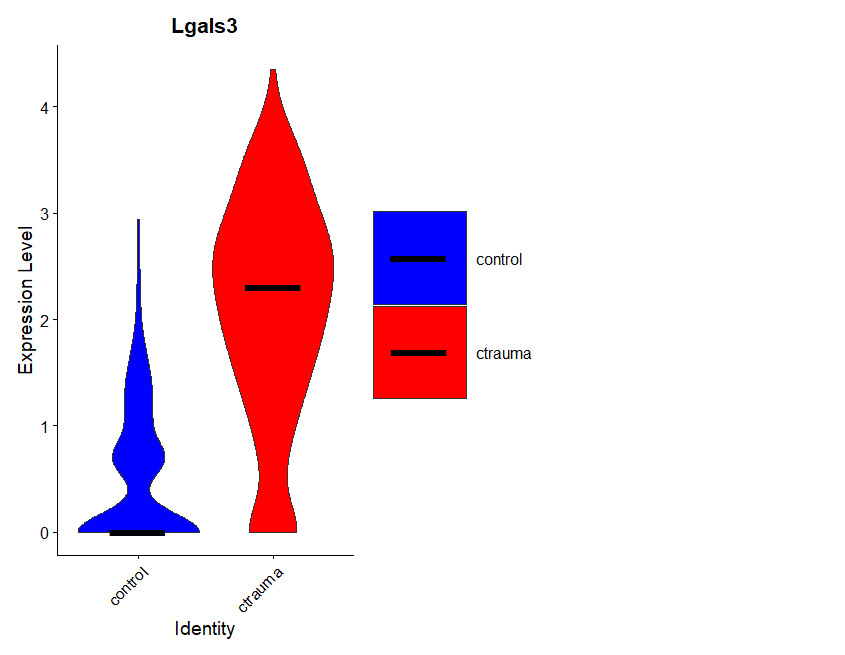

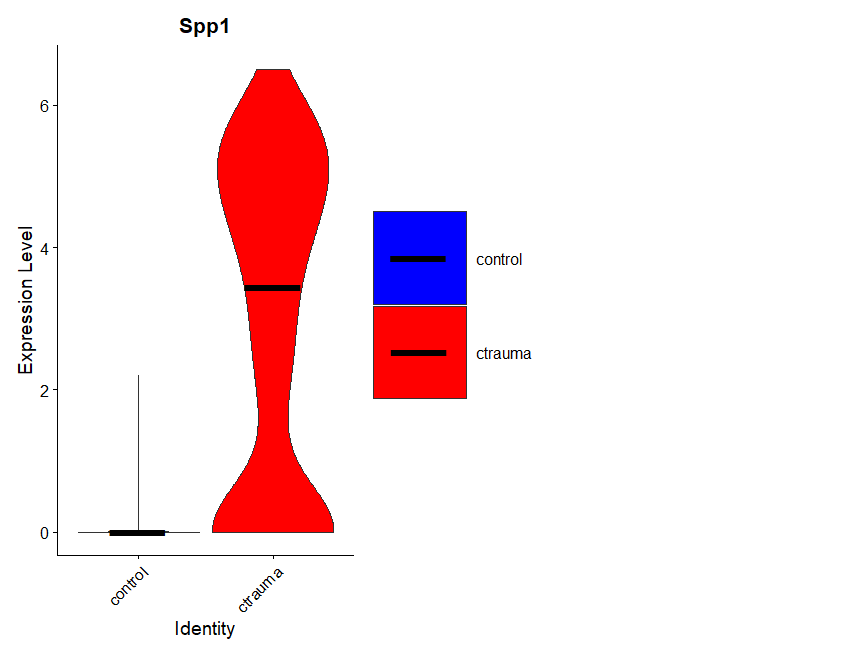


**Mono/Mac Express Hub Genes**

**a**


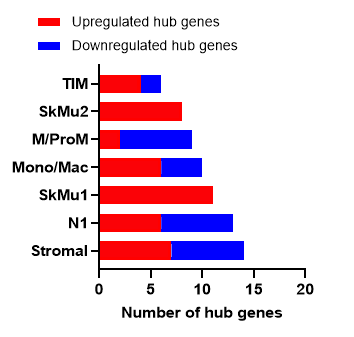


**b**

**Figure S14. Related to Figure 7.** Expression patterns of top 15 hub genes after polytrauma. (a) Violin plots showing increased *Itgb2, Tyrobp, Lgals3,* and *Spp1* expression in Mono/Mac cells after polytrauma compared to control, with median expression level shown by center line. (b) Barplot showing differential regulation of hub genes across cell types following polytrauma.


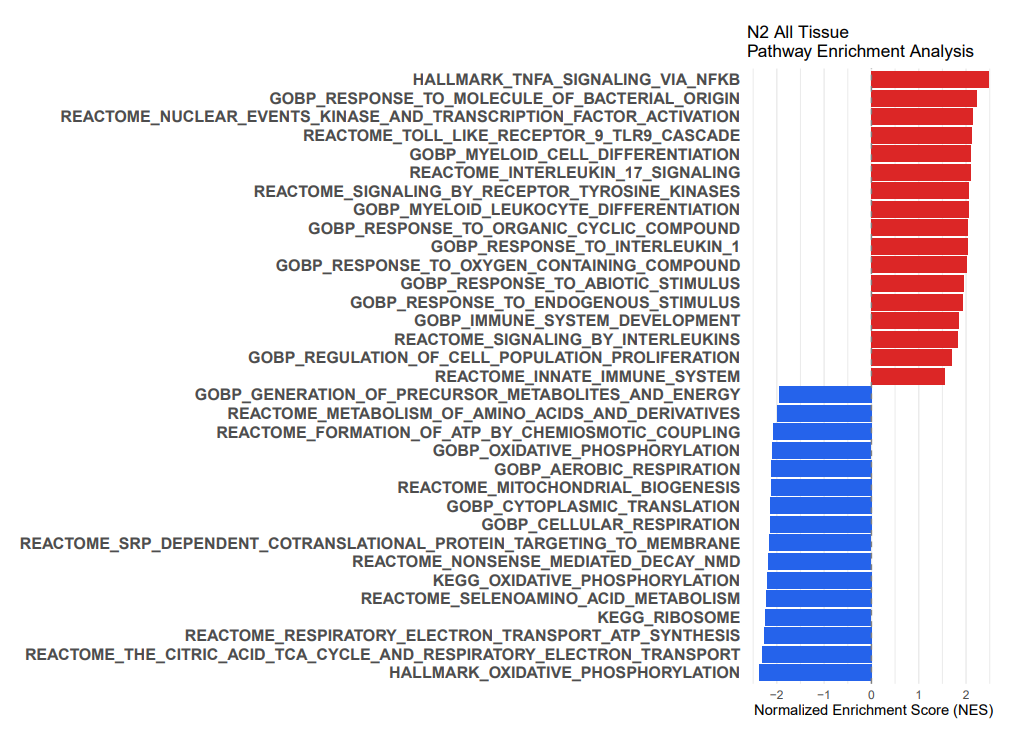

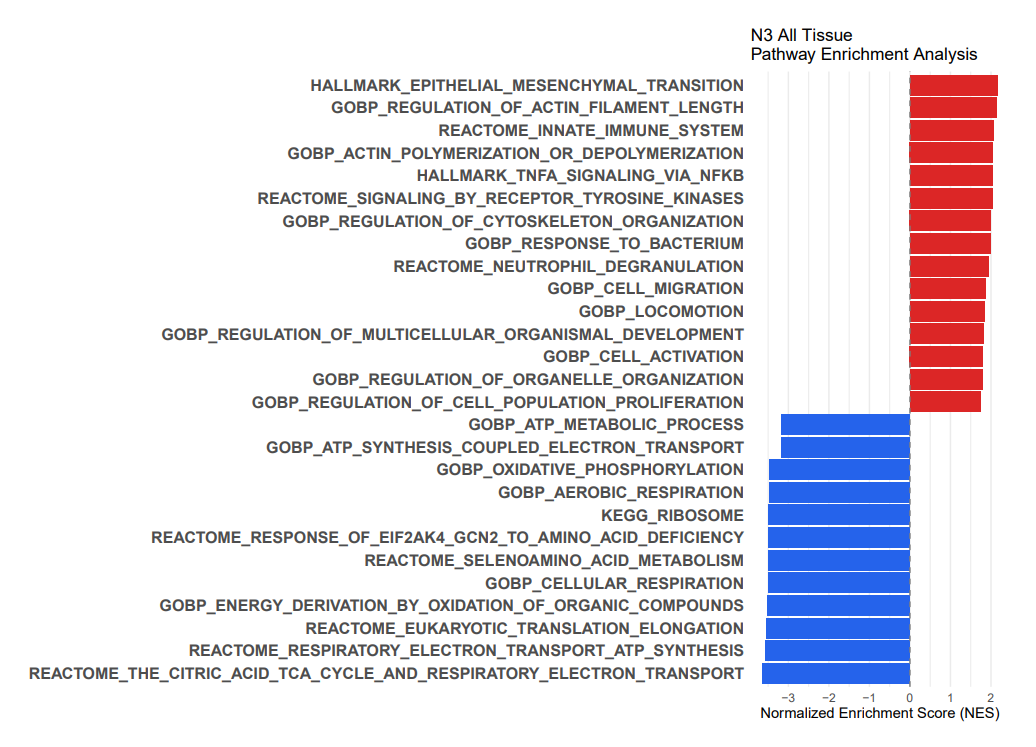


**b**

**a**

**Figure S15. Related to Figure 7.** GSEA pathways enriched in neutrophil clusters from the all-tissue integrated dataset. (a) Pathways regulated in N2 cluster comparing polytrauma to control baseline. (b) Pathways regulated in N3 cluster comparing polytrauma to control baseline. Normalized Enrichment Score (NES) indicates pathway changes in polytrauma relative to control: positive NES shows upregulation; negative NES shows downregulation (p-value adjusted < 0.05).


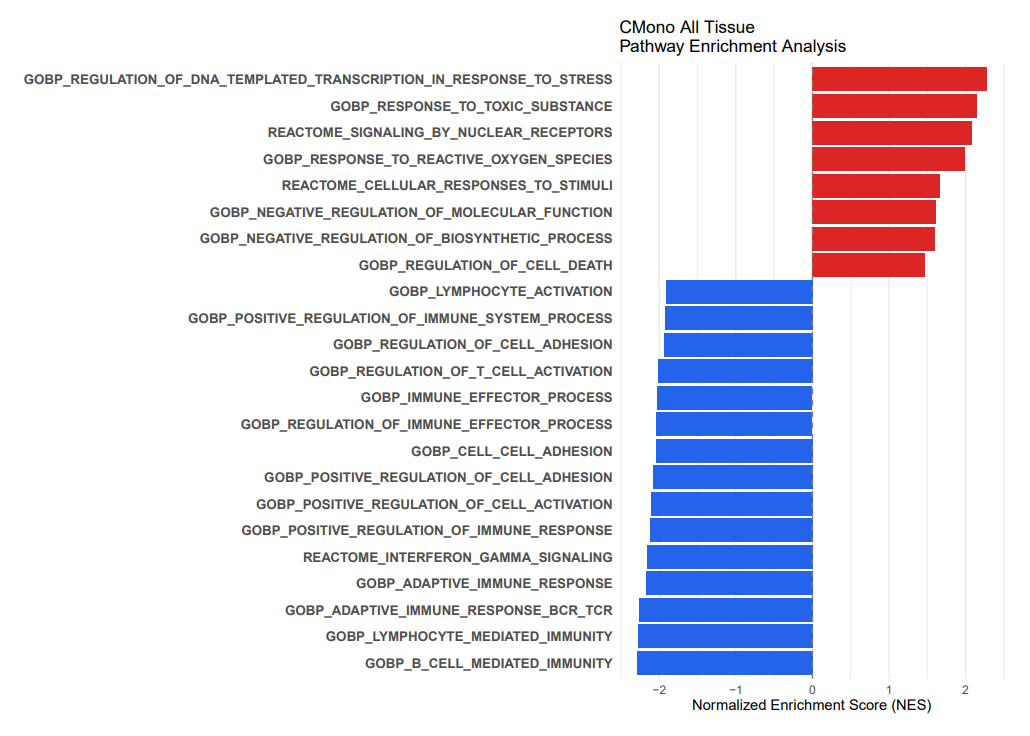


**Figure S16. Related to Figure 7.** GSEA pathways enriched in classical monocytes from the all-tissue integrated dataset. Pathways calculated by comparing polytrauma to control baseline. Normalized Enrichment Score (NES) indicates pathway changes in polytrauma relative to control: positive NES shows upregulation; negative NES shows downregulation (p-value adjusted < 0.05).


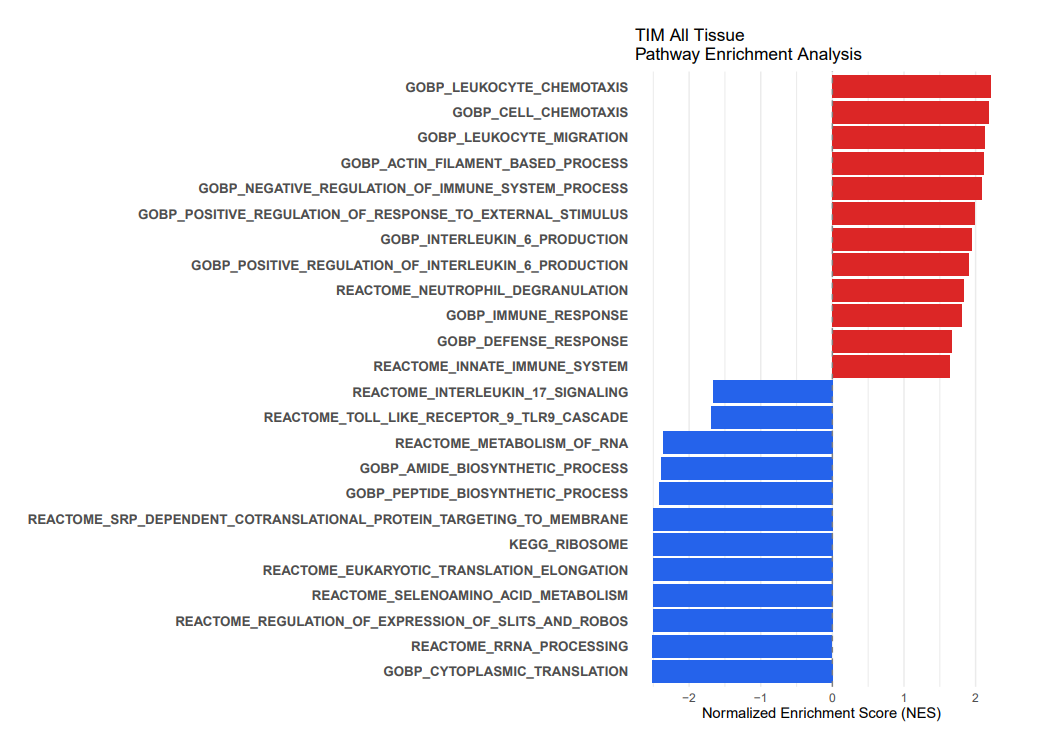


**a**


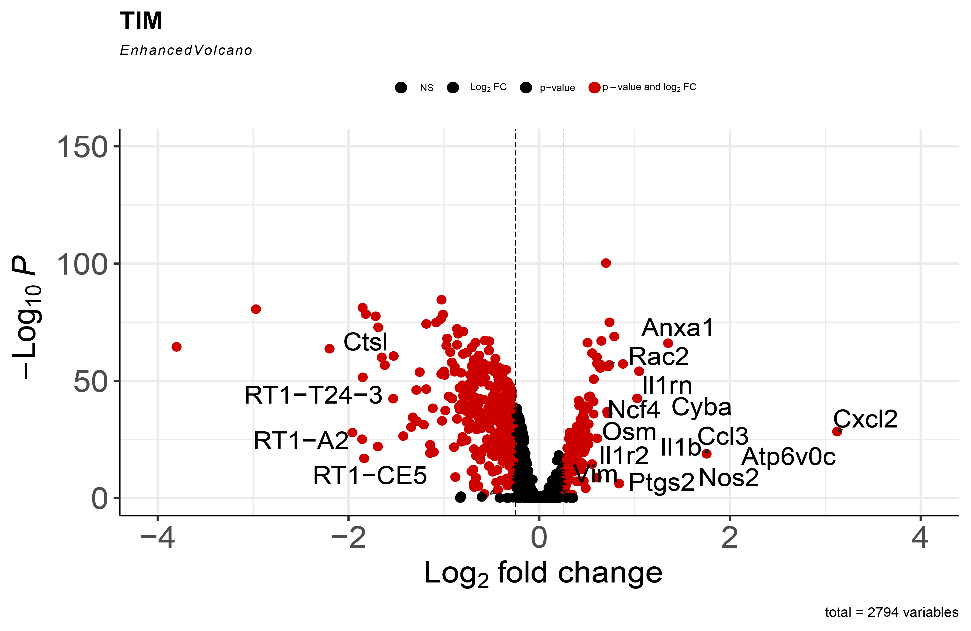

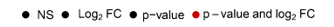


**b**

**c**


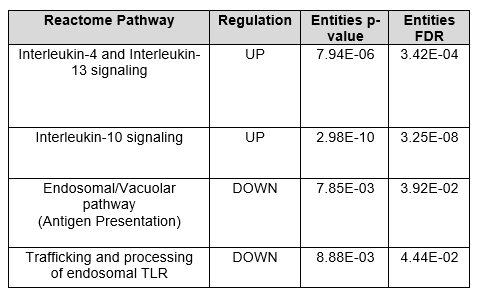


**Figure S17. Related to Figure 7**. Characterization of TIM cells through GSEA pathways, DEGs, and associated pathways from the all-tissue integrated dataset. (a) GSEA pathways enriched in TIM cells by comparing polytrauma to control (baseline). Normalized Enrichment Score (NES) indicates pathway changes in polytrauma relative to control: positive NES shows upregulation; negative NES shows downregulation (p-value adjusted < 0.05). (b) DEGs upregulated by TIM cells in polytrauma compared to control. (c) ORA analysis identified immune-specific pathways that are either up- or downregulated by TIM cells. ORA was conducted on the top 200 most up- and downregulated DEGs, and significant immune system pathways were determined. The significance cutoff for the pathways was set at p-value < 0.01 and false discovery rate (FDR) < 0.05.


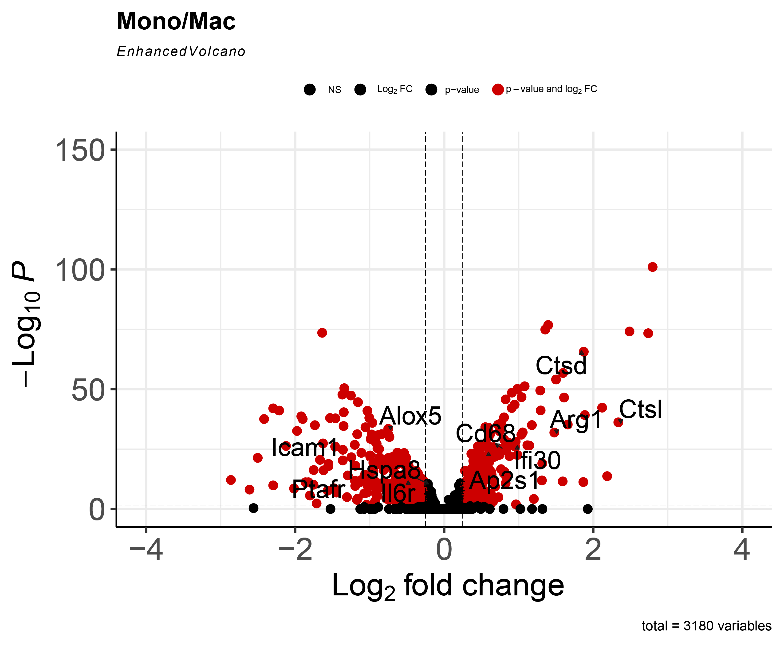

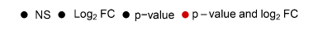


**b**

**c**


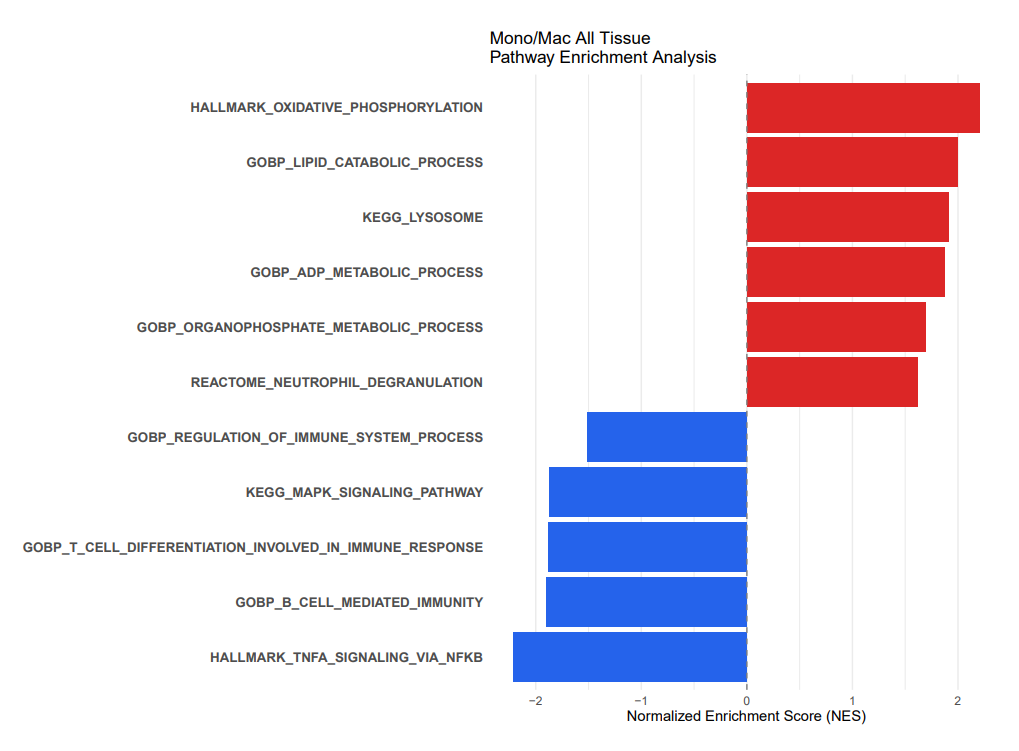


**a**


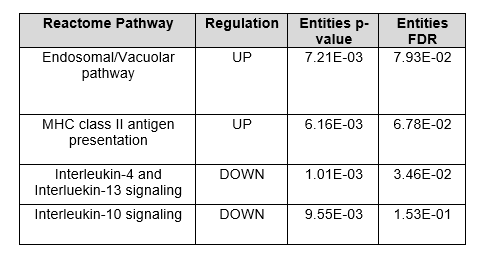


**Figure S18. Related to Figure 7**. Characterization of Mono/Mac cells through GSEA pathways, DEGs, and associated pathways from the all-tissue integrated dataset. (a) GSEA pathways enriched in Mono/Mac cells by comparing polytrauma to control (baseline). Normalized Enrichment Score (NES) indicates pathway changes in polytrauma relative to control: positive NES shows upregulation; negative NES shows downregulation (p-value adjusted < 0.05). (b) DEGs upregulated by Mono/Mac cells in polytrauma compared to control. (c) ORA analysis identified immune-specific pathways that are either up- or downregulated by Mono/Mac cells. ORA was conducted on the top 200 most up- and downregulated DEGs, and significant immune system pathways were determined. The significance cutoff for the pathways was set at p-value < 0.01 and false discovery rate (FDR) < 0.05


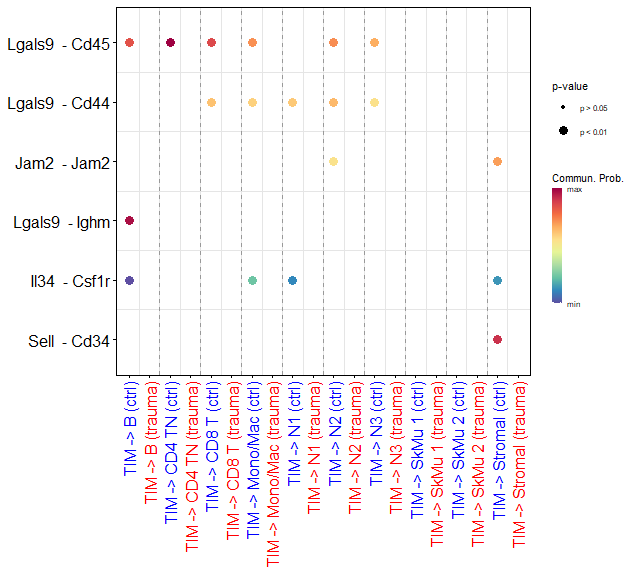

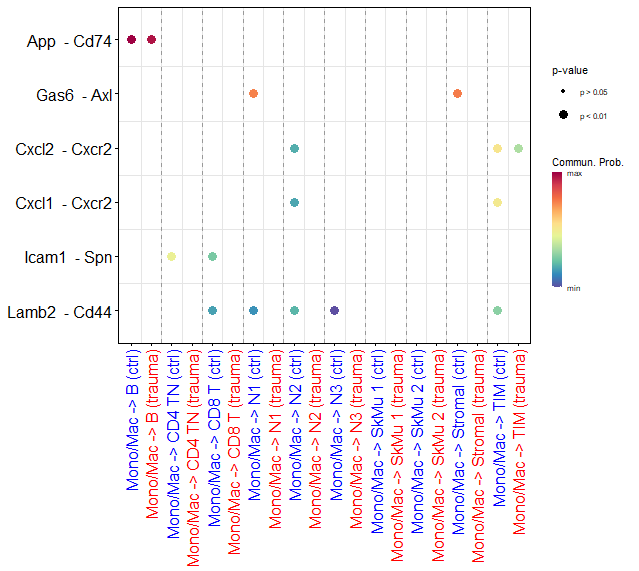


**a**

**b**

**Figure S19. Related to Figure 7.** Dot plot showing downregulated ligand-receptor interactions in polytrauma compared to control conditions. (a) Downregulated interactions initiated by TIM cells. (b) Downregulated interactions initiated by Mono/Mac cells.
